# Systematic early versus late mobilization or standard early mobilization in mechanically ventilated adult ICU patients: systematic review and meta-analysis

**DOI:** 10.1186/s13054-020-03446-9

**Published:** 2021-01-06

**Authors:** Dominik Menges, Bianca Seiler, Yuki Tomonaga, Matthias Schwenkglenks, Milo A. Puhan, Henock G. Yebyo

**Affiliations:** 1 Department of Epidemiology, Epidemiology, Biostatistics and Prevention Institute (EBPI), University of Zurich, Hirschengraben 84, 8001 Zurich, Switzerland; 2 Faculty of Medicine (MeF), University of Zurich, Pestalozzistrasse 3, 8091 Zurich, Switzerland; 3 Institute of Pharmaceutical Medicine (ECPM), University of Basel, Klingelbergstrasse 61, 4056 Basel, Switzerland

**Keywords:** Early mobilization, Rehabilitation, Intensive care, Critical care, ICU, Physical therapy, Mechanical ventilation, Systematic review, Meta-analysis

## Abstract

**Background:**

This systematic review and meta-analysis aimed to determine the effectiveness of systematic early mobilization in improving muscle strength and physical function in mechanically ventilated intensive care unit (ICU) patients.

**Methods:**

We conducted a two-stage systematic literature search in MEDLINE, EMBASE and the Cochrane Library until January 2019 for randomized controlled trials (RCTs) examining the effects of early mobilization initiated within 7 days after ICU admission compared with late mobilization, standard early mobilization or no mobilization. Priority outcomes were Medical Research Council Sum Score (MRC-SS), incidence of ICU-acquired weakness (ICUAW), 6-min walk test (6MWT), proportion of patients reaching independence, time needed until walking, SF-36 Physical Function Domain Score (PFS) and SF-36 Physical Health Component Score (PCS). Meta-analysis was conducted where sufficient comparable evidence was available. We evaluated the certainty of evidence according to the GRADE approach.

**Results:**

We identified 12 eligible RCTs contributing data from 1304 participants. Two RCTs were categorized as comparing systematic early with late mobilization, nine with standard early mobilization and one with no mobilization. We found evidence for a benefit of systematic early mobilization compared to late mobilization for SF-36 PFS (MD 12.3; 95% CI 3.9–20.8) and PCS (MD 3.4; 95% CI 0.01–6.8), as well as on the proportion of patients reaching independence and the time needed to walking, but not for incidence of ICUAW (RR 0.62; 95% CI 0.38–1.03) or MRC-SS. For systematic early compared to standard early mobilization, we found no statistically significant benefit on MRC-SS (MD 5.8; 95% CI − 1.4 to 13.0), incidence of ICUAW (RR 0.90; 95% CI 0.63–1.27), SF-36 PFS (MD 8.1; 95% CI − 15.3 to 31.4) or PCS (MD − 2.4; 95% CI − 6.1 to 1.3) or other priority outcomes except for change in 6MWT from baseline. Generally, effects appeared stronger for systematic early compared to late mobilization than to standard early mobilization. We judged the certainty of evidence for all outcomes as very low to low.

**Conclusion:**

The evidence regarding a benefit of systematic early mobilization remained inconclusive. However, our findings indicate that the larger the difference in the timing between the intervention and the comparator, the more likely an RCT is to find a benefit for early mobilization.

*Study Registration*: PROSPERO (CRD42019122555).

## Background

Patients in intensive care units (ICUs) frequently suffer from ICU-acquired weakness (ICUAW) and lasting physical and neurocognitive impairment, resulting in difficulties in achieving full functionality in their social and professional lives [1–3]. As a consequence, ICU stays are associated with a reduced quality of life as well as increased utilization of medical care, costs and mortality [2, 3].

The systematic early mobilization of ICU patients is commonly advocated as an intervention to improve patient outcomes [1, 4] and is part of various clinical practice guidelines [5–9]. There is evidence from several studies that early mobilization may improve physical function, decrease the risk of acquiring ICUAW or delirium and shorten the time to weaning from mechanical ventilation [10–13]. However, some systematic reviews found no or inconclusive evidence for a benefit [14, 15]. It is not fully clear how the inconsistency in effects between studies arises. While heterogeneity in study populations and modality or intensity of study interventions may play a role [15], the timing of early mobilization has been discussed as an important factor for the effectiveness of the intervention, with earlier interventions showing greater benefit [4]. However, the definition of standard care is not consistent between trials and may have changed over time as early mobilization was increasingly adopted in clinical practice. Thus, standard care may involve mobilization approaches that are also provided early, but less systematically [4, 15]. This may complicate the evaluation of the effects of early mobilization.

In this systematic review and meta-analysis, we aimed to determine the effectiveness of systematic early mobilization in mechanically ventilated adult ICU patients, while explicitly considering the timing of the delivery of the comparator intervention.

## Methods

We conducted this systematic review in line with the Preferred Reporting Items for Systematic Reviews and Meta-Analyses (PRISMA) and Cochrane Collaboration recommendations [16, 17]. A protocol was registered a priori on PROSPERO (CRD42019122555).

### Eligibility criteria

#### Population

We included studies conducted in adult ICU patients (aged ≥ 18 years) requiring invasive or non-invasive mechanical ventilation at enrollment or during the ICU stay. We excluded studies that enrolled relevant proportions (≥ 10%) of patients with burn injuries, neurological conditions or transplant patients, as well as studies conducted in postoperative patients requiring ventilation for less than 24 h on average, as we considered these patients to have different needs or be at higher risk for adverse events than other ICU patients.

#### Intervention

The experimental intervention of interest was *systematic early mobilization*, which we defined as any physical or occupational therapy targeting muscle activation, initiated within 7 days after ICU admission and performed according to a clearly defined protocol or specific clinical criteria in all eligible patients. Neurocognitive interventions, speech therapy and ICU diary keeping were considered eligible as part of an early rehabilitation approach including systematic mobilization. Studies examining interventions primarily targeted at preventing pressure ulcers or joint stiffness, or respiratory therapy alone were not included.

#### Comparators

Based on a priori-defined criteria, eligible comparators were categorized as: (i) *late mobilization* (i.e., mobilization initiated 7 days or more after ICU admission), (ii) *standard early mobilization* (i.e., mobilization initiated within 7 days but less systematically, as outlined above) or (iii) *no mobilization* (i.e., sham intervention or no rehabilitative intervention).

#### Outcomes

As part of a comprehensive assessment, we prespecified multiple primary outcomes related to muscle strength and functional mobility and secondary outcomes related to quality of life, mortality, length of stay and safety (see Additional file 1). Follow-up time points considered included ICU discharge, hospital discharge, as well as 3, 6 and 12 months after hospital discharge. Out of all outcomes, the most clinically important and patient-relevant outcomes were prioritized by four ICU experts involved as stakeholders in this project without prior knowledge of the data. Here, we primarily report on these priority outcomes, which include the Medical Research Council Sum Score (MRC-SS) at ICU discharge, proportion of patients developing ICUAW during hospitalization, 6-min walk test (6MWT) performance, time needed until walking for the first time, proportion of patients returning to independence from assistance, SF-36 Physical Function Domain Score (PFS) and SF-36 Physical Health Component Summary Score (PCS) at 6 months after discharge.

### Study types

Only randomized controlled trials (RCTs) published in English, German, French or Italian language were included. We did not consider observational evidence as we assumed a high probability of confounding by indication and differences in the provision of early mobilization between patients in a non-standardized, non-randomized setting.

### Information sources and search strategy

To identify relevant studies, we followed a two-stage systematic search process based on previously published high-quality systematic reviews. In the first stage, we systematically searched the MEDLINE and Cochrane Library databases for relevant systematic reviews published between 2015 and 2019. We assessed the identified systematic reviews in full text for eligibility and selected high-quality systematic reviews based on the Assessing the Methodology of Systematic Reviews (AMSTAR 2) assessment checklist [18]. The selected high-quality systematic reviews were then used as a basis to identify potentially eligible RCTs. All records identified in these reviews were included in the full-text assessment in the second stage of our systematic review.

In the second stage, we performed a systematic follow-up search in the MEDLINE, EMBASE, CINAHL and CENTRAL databases to identify more recently published studies. We adopted the search strategies of the high-quality reviews, additionally applying the Cochrane sensitivity and precision-maximizing RCT filter [19]. Each search was conducted for a timeframe starting two months prior to the last search in the respective review up to January 17, 2019, to account for a potential lag in the indexing of publications (see Additional file 1 for detailed search strategies). Additional references were identified through bibliographies of included studies and registry records. We screened the title and abstract of all records retrieved through the update searches and pooled potentially eligible records with the records retrieved from the high-quality systematic reviews. After deduplication, we assessed the pooled references in full text to select eligible studies. All study selection processes were carried out independently and in duplicate by three reviewers. Disagreements were resolved by consensus with an experienced senior reviewer.

### Data extraction

We extracted information regarding the study design, study population characteristics, intervention and comparator details (i.e., modality, timing, frequency, duration), measured outcomes and follow-up. Where reporting of intervention, comparator or results was insufficient to allow judgments about the categorization of studies, we consulted study protocols and contacted authors for additional information. Data extraction was performed in duplicate by three reviewers.

### Risk of bias assessment

We assessed the risk of bias of included RCTs using the Cochrane risk of bias tool [17, 20] and evaluated study-level bias as recommended by the Agency for Healthcare Research and Quality (AHRQ) [21]. As blinding of personnel is commonly not possible in the context of rehabilitative interventions, this domain was not considered for the study-level assessment.

### Data synthesis

We primarily used a narrative synthesis due to the high heterogeneity between RCTs, measured outcomes and follow-up time points. As we considered the comparator interventions to be a major source of heterogeneity, we report results stratified by comparator category (i.e., *late mobilization*, *standard early mobilization* or *no mobilization*). Studies were categorized according to the timing and the nature of the comparator intervention. Studies in which the comparator did not meet the definition of early mobilization were assigned to the *late mobilization* category. Studies in which the comparator was also administered early, but in a less protocol-driven and consistent manner, according to less strict criteria or not in all eligible patients, were assigned to the *standard early mobilization* category. Studies that could not be categorized with respect to the timing of the comparator were also assigned to the standard early mobilization category, in order to enable separate evaluation of studies in which a clear timing difference between groups was present (i.e., comparing early with late mobilization). Studies with a sham procedure or no rehabilitative intervention as comparator were assigned to the *no mobilization* category.

We conducted pairwise fixed- and random-effects meta-analyses for outcomes that were reported by at least three studies. Studies reporting median and interquartile range (IQR) only were not included in the meta-analyses. We report risk ratios (RRs) for dichotomous outcomes and mean differences (MD) for continuous outcomes. Study heterogeneity was assessed visually using forest plots and statistically using the *I*^2^-statistic. We further conducted sensitivity analyses to explore heterogeneity. We planned to conduct heterogeneity assessment based on predefined factors (continuation of the intervention post ICU discharge, intervention type, study population characteristics, study-level risk of bias) and to assess small study effects using funnel plots and Egger's test, where appropriate. However, the number of studies for each reported outcome was too low to allow a meaningful assessment. Preplanned subgroup analyses based on age and length of ICU stay were not possible because no separate data were reported for these populations. We used R (version 3.5.2) for all statistical analyses.

### Confidence in evidence

We assessed the confidence in the evidence using the Grading of Recommendations Assessment, Development, and Evaluation (GRADE) approach for the priority outcomes [22].

## Results

### Study selection

**Fig. 1:**
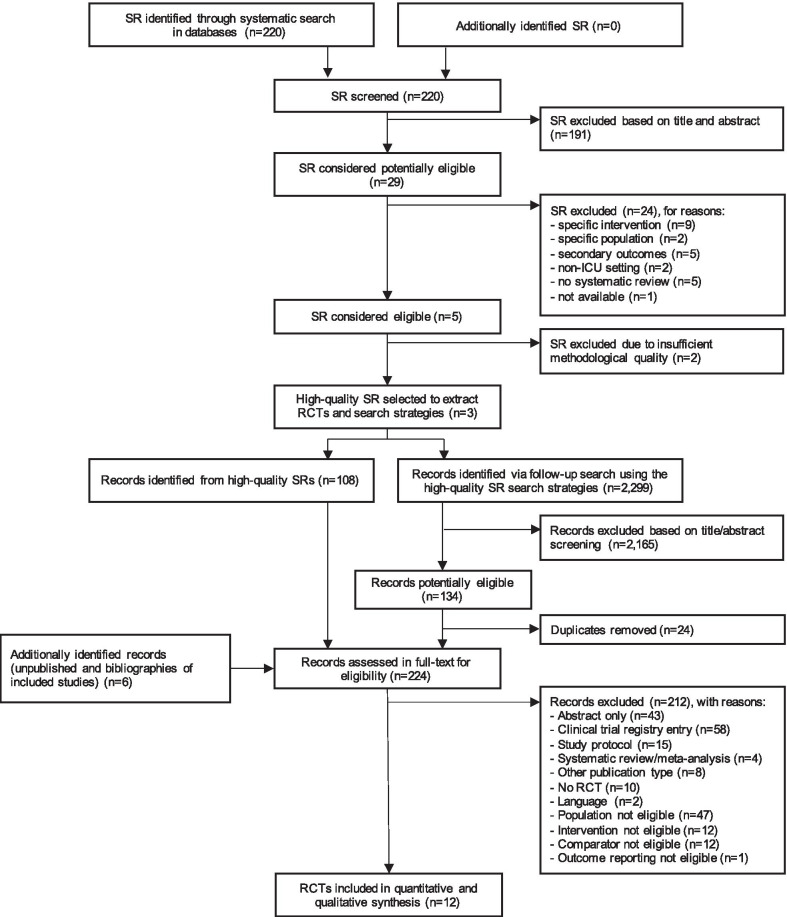
PRISMA flow diagram of the study selection process. *ICU* intensive care unit, *SR* systematic review, *RCT* randomized controlled trial

In the first stage of the literature search, we found three high-quality systematic reviews published between 2015 and 2019 [12, 14, 15], through which we identified 108 references. In the second stage, the systematic update search yielded further 2,299 records, and six references were identified through bibliographies from relevant publications. Twelve studies were finally included in the qualitative and quantitative analysis [23–34]. Figure 1 shows the study selection process and the main reasons for exclusion at the different stages.

### Study characteristics

The included studies provided data from 679 people randomized to systematic early mobilization and 625 people receiving one of the comparators. We categorized two studies as comparing systematic early against late mobilization [23, 32] and the majority of studies (9 out of 12) as comparing systematic early against standard early mobilization [24–29, 31, 33, 34]. Six studies did not report information on the time from ICU admission to first mobilization in the intervention group [24, 25, 27–29, 33]. Information about the timing difference between intervention and comparator group was unavailable for six studies [25–29, 33], which were thus included in the *standard early mobilization* category. One study was categorized as comparing systematic early mobilization against no mobilization, but contributed data to secondary outcomes reported in Additional file 3 only [30].

An overview of the included studies, study participant characteristics and interventions is provided in Tables 1 and 2. There was considerable heterogeneity in the baseline characteristics of participants in terms of gender, age and disease severity, both between studies and between intervention and comparator groups within studies. While most studies included a diverse mix of diagnoses, three studies focused on specific populations such as cardiothoracic surgery [29, 30] or sepsis patients [28]. While interventions primarily involved physical therapy, one study additionally investigated combined physical and cognitive therapy in one of the intervention groups [26]. Two studies involved occupational therapy [23, 26] and two included neuromuscular electro-stimulation [28, 30]. None of the studies involved speech therapy or ICU diary keeping in addition to early mobilization.

**Table 1:** Summary of included studies and study participants

Study	Country, timeframe	Population	Group	No. of participants	Female n (%)	Age in years mean (SD)/median (IQR)	APACHE II score mean (SD)/median (IQR)	Patient admission diagnoses
*(i) Systematic early vs. late mobilization*								
Schweickert et al. [23]	USA 2005–2007	Adult ICU patients, mechanically ventilated < 72 h, independent at baseline	Comparator	55	23 (41.8)	54.4 (46.5–66.4)	19.0 (13.3–23.0)	Lung injury (56%), COPD exacerbation (10%), acute exacerbation of asthma (9%), sepsis (15%), hemorrhage (3%), malignancy (3%), other (5%)
			Intervention	49	29 (59.2)	57.7 (36.3–69.1)	20.0 (15.8–24.0)	
Morris et al. [32]	USA 2009–2014	Adult ICU patients, acute respiratory failure requiring mechanical ventilation	Comparator	150	82 (54.7)	58 (14)	75.0 (27.0)^b^	Acute respiratory failure (98%), coma (2%)
			Intervention	150	84 (56.0)	55 (17)	76.0 (26.0)^b^	
*(ii) Systematic early vs. standard early mobilization*								
Dantas et al. [24]	Brazil 2009–2011	Adult ICU patients on mechanical ventilation, adequate cardiovascular and respiratory reserve	Comparator	14	10 (71.4)	50.4 (20.5)	21.1 (7.2)	Acute respiratory failure (46%), pneumonia (14%), cardiomyopathy (0%), collagenosis (4%), postoperative after thoraco-abdominal surgery (11%), acute myocardial infarction (7%), leptospirosis (4%), acute renal insufficiency (4%), pulmonary tuberculosis (7%), neoplasms (4%)
			Intervention	14	7 (50.0)	59.1 (15.2)	23.7 (8.5)	
Denehy et al. [25]	Australia 2007–2009	Adult ICU patients, ICU length of stay ≥ 5 days	Comparator	76	31 (40.8)	60.1 (15.8)	20.7 (7.7)	Pneumonia (23%), cardiac (15%), cardiac arrest (7%), cardiac surgery (30%), other surgery (21%), liver disease/transplant (14%), sepsis (11%), renal (5%), other (7%)
			Intervention	74	24 (32.4)	61.4 (15.9)	19.0 (6.0)	
Brummel et al. [26]^a^	USA 2011–2012	Adult ICU patients, respiratory failure and/or septic, cardiogenic or hemorrhagic shock, critically ill for < 72 h	Comparator	22	14 (63.6)	60 (51–69)	27.0 (17.5–31.0)	Sepsis/ARDS/pneumonia (60%), abdominal surgery (15%), other surgery (3%), airway protection (9%), cirrhosis/GI bleeding (5%), CHF/arrhythmia/cardiogenic shock (2%), other (6%)
			Intervention (1)	22	9 (40.9)	62 (48–67)	21.5 (20.0–28.8)	
			Intervention (2)	43	15 (34.9)	62 (54–69)	25.0 (19.5–29.5)	
Dong et al. [27]	China 2010–2012	Adult ICU patients, mechanically ventilated between 48–72 h with expected ventilation of ≥ 1 week, clear consciousness, cardiovascular and respiratory stability	Comparator	30	10 (33.3)	55.5 (16.2)	16.0 (4.1)	Abdominal infections (18%), ARDS (32%), sepsis (7%), severe acute pancreatitis (15%), pneumonia (23%), COPD exacerbation (5%)
			Intervention	30	9 (30.0)	55.3 (16.1)	15.0 (4.2)	
Kayambu et al. [28]	Australia 2010–2012	Adult ICU patients, mechanically ventilated ≥ 48 h, diagnosis of sepsis or septic shock	Comparator	24	10 (41.7)	65.5 (37–85)	27.0 (6.8)	Sepsis (100%)
			Intervention	26	8 (30.8)	62.5 (30–83)	28.0 (7.6)	
Dong et al. [29]	China 2012–2015	Adult patients, prolonged mechanical ventilation > 72 h, eligible for coronary artery bypass surgery	Comparator	53	31 (58.5)	60.2 (15.1)	17.2 (4.3)	Coronary artery bypass surgery (100%)
			Intervention	53	33 (62.3)	62.6 (12.8)	16.3 (4.2)	
Hodgson et al. [31]	Australia/New Zealand 2013–2014	Adult ICU patients, mechanically ventilated within 72 h of ICU admission	Comparator	21	12 (57.1)	53 (15)	15.9 (6.9)	N/A
			Intervention	29	8 (25.9)	64 (12)	19.8 (9.8)	
Schaller et al. [33]	USA/Germany 2011–2015	Adult surgical ICU patients, mechanically ventilated for less than 48 h and for at least further 24 h, functionally independent at baseline	Comparator	96	35 (36.5)	64 (45–76)	17 (11–22)	Visceral surgery (27%), vascular surgery (17%), ENT and ophthalmological surgery (10%), transplant surgery (4%), neurosurgery (3%), orthopedic surgery (3%), thoracic surgery (3%), gynecological surgery (2%), urological surgery (1%), plastic surgery (1%), medical or neurological diagnosis (6%), trauma (26%)
			Intervention	104	39 (37.5)	66 (48–73)	16 (12–22)	
Eggmann et al. [34]	Switzerland 2012–2016	Adult ICU patients, expected to stay on mechanical ventilation for at least 72 h, independent before critical illness	Comparator	57	16 (28.1)	63 (15)	23.0 (7.0)	Cardiac surgery (18%), neurology/neurosurgery (8%), other surgery (12%), gastroenterology (12%), trauma (4%), respiratory insufficiency (22%), hemodynamic insufficiency (23%), other (2%)
			Intervention	58	22 (37.9)	65 (15)	22.0 (8.0)	
*(iii) Systematic early vs. no mobilization*								
Fischer et al. [30]	Austria 2011–2012	Patients with cardiothoracic surgery, anticipated ICU stay of ≥ 48 h	Comparator	27	7 (25.9)	69.7 (13.1)	N/A	Cardiothoracic surgery (100%)
			Intervention	27	9 (33.3)	63.3 (15.5)	N/A	

*APACHE* Acute Physiologic Assessment and Chronic Health Evaluation, *ARDS* acute respiratory distress syndrome, *COPD* chronic obstructive pulmonary disease, *ICU* intensive care unit, *IQR* interquartile range, *N/A* not available, *SD* standard deviation

^a^Three-arm trial

^b^APACHE III score

**Table 2:** Details on study interventions and comparators

Study	Group	Intervention description	Time to first intervention	Intervention frequency	Intervention duration	Intervention continuation
*(i) Systematic early vs. late mobilization*						
Schweickert et al. [23]	Comparator	Standard care: therapy as ordered by the primary care team	Median 7.4 days (IQR 6.0–10.9) after intubation	N/A	Median 0.0 h (IQR 0.0–0.0) per day during ventilation; 0.2 h (IQR 0.0–0.4) per day without ventilation	Not specified
	Intervention	Passive range of motion, active range of motion, including bed mobility exercises, activities of daily living and other exercises increasing independency, transfer training (sit to stand, bed to chair, bed to commode), pre-gait exercises, walking	Median 1.5 days (IQR 1.0–2.1) after intubation	Once daily	Median 0.3 h (IQR 0.2–0.5) per day during ventilation; 0.2 h (IQR 0.1–0.3) per day without ventilation	Until hospital discharge
Morris et al. [32]	Comparator	Usual care: weekday physical therapy when ordered by the team	Median 7 days (IQR 4–10) after ICU admission	N/A	N/A	Not specified
	Intervention	Passive range of motions, physical therapy and progressive resistance exercises	Median 1 days (IQR 0–2) after ICU admission	3 times daily, 7 days a week	N/A	Until hospital discharge
*(ii) Systematic early vs. standard early mobilization*						
Dantas et al. [24]	Comparator	Conventional physical therapy: passive mobilization of the four limbs five times a week and active-assisted exercises according patients’ improvements	N/A (all participants completed first session within 48 h after admission^b^)	5 times per week	N/A	Until ICU discharge
	Intervention	Passive stretching and mobilization of the four limbs, positioning of the joints, active assisted exercises of the four limbs, transfer from lying to sitting position, active resistive exercises (against gravity or with weight) of upper limbs, cycle ergometry for lower limbs, transfer from sitting to chair, orthostatic posture, counter-resistance exercise on upper limbs, balance exercises, walking	N/A (all participants completed first session within 48 h after admission^b^)	Twice daily	N/A	Until ICU discharge
Denehy et al. [25]	Comparator	Usual care: active bed exercises, sitting out of bed, marching or walking	N/A (enrollment earliest at day 5^b^)	N/A	N/A	Until hospital discharge
	Intervention	ICU: arm and leg active and active resistance movements, moving from sitting to standing, marching in place; ward: cardiovascular, progressive resistance strength training and functional exercise; Outpatient: cardiovascular, progressive resistance strength training and functional exercise	N/A (enrollment earliest at day 5)	Once daily while ventilated; twice daily after weaning	15 min per day in mechanically ventilated; 2 times 15 min per day in weaned; 2 times 30 min per day on ward; 2 times 60 min per week as outpatients for 8 weeks	Beyond hospital stay
Brummel et al. [26]^a^	Comparator	Usual care: existing ICU mobility protocol	Median 3 days (IQR 2–6) after enrollment	1–2 times per week	N/A	Not specified
	Intervention (1)	Physical therapy: passive range of motion, sit at the edge of bed, stand, walk, activities of daily living	Median 1 days (IQR 1–1) after enrollment	Once daily	Median 15 min (IQR 10–20) for physicians & nurses; median 23 min (IQR 16–26) for physiotherapy	Until hospital discharge
	Intervention (2)	Cognitive plus physical therapy: same as in physical therapy only + orientation, digit span forward, matric puzzle, real world, digit span reverse, noun list recall, letter-number sequences, pattern recognition	Median 1 days (IQR 1–1) after enrollment, 3 days (IQR 2–4) after ICU admission	Cognitive therapy twice daily; Physical therapy once daily	Cognitive therapy median 20 min; Physical therapy median 15 min for physicians & nurses, median 23 min for physiotherapy	Beyond hospital stay
Dong et al. [27]	Comparator	Control (not further described)	N/A	N/A	N/A	Not specified
	Intervention	Heading up actively, transferring from supine to sitting position, to sitting at the edge of bed, to sitting in a chair, from sitting to standing, walking bedside	N/A	Twice daily	Tailored depending on the condition of patients	Until hospital discharge
Kayambu et al. [28]	Comparator	Standard care: same as in intervention group but less	N/A (4% completed first session within 48 hours^b^)	N/A	N/A	Until ICU discharge
	Intervention	NMES, passive range of motion, active range of motion, active resistance exercises, sitting up in bed, sitting out of bed, sit to stand, marching on the spot, sitting and standing balance exercises, arm or leg ergometry, tilt table therapy, ambulation	N/A (46% completed first session within 48 hours^b^)	1–2 times daily	30 min	Until ICU discharge
Dong et al. [29]	Comparator	Therapy only after ICU	N/A	N/A	N/A	Not specified
	Intervention	Head up, transferring from supine to sitting position, sitting at the edge of bed, sitting in a chair, transferring from sitting to standing, walking along the bed	N/A (100% completed first step in first session)	Twice daily	N/A	Not specified
Hodgson et al. [31]	Comparator	Passive movements, same equipment would have been available	Median 4 days (IQR 3–5)^b^	Once daily	5–10 min per day	Until ICU discharge
	Intervention	Functional activities, active bed exercises, comprising walking as long as possible, standing as long as possible, balance exercises, sitting in or out of bed, sitting balance, sit to stand, rolling	Median 3 days (IQR 2–4)	Once daily	30–60 min depending on the condition of patients	Until ICU discharge
Schaller et al. [33]	Comparator	In line with the individual centers’ practice guidelines for mobilization and physical therapy	N/A	N/A	N/A	Not specified
	Intervention	Mobilization according to mobility algorithm: passive range of motion, sitting, standing, ambulation. Interprofessional mobility goal setting and identification of barriers	N/A	Once daily	Tailored depending on the condition of patients	Not specified
Eggmann et al. [34]	Comparator	Usual care as per the European standard physiotherapy and individually tailored but subject to medical prescription	Median 2.2 days (IQR 1.5–2.9) after ICU admission	Once daily, 5 days per week	Median 18 min (IQR 14–21)	Until hospital discharge
	Intervention	Motor-assisted bed-cycle, resistant training for upper and lower limbs, sitting on bedside, sitting in a chair, standing, walking	Median 2.0 days (IQR 1.4–2.8) after ICU admission	Up to 3 times daily, 7 days per week	Median 25 min (IQR 19.5–27)	Not specified
*(iii) Systematic early vs. no mobilization*						
Fischer et al. [30]	Comparator	Sham NMES	First postoperative day	Twice daily, 7 days per week	30 min per session (60 min daily)	Until ICU discharge
	Intervention	NMES	First postoperative day	Twice daily, 7 days per week	30 min per session (60 min daily)	Until ICU discharge

*ICU* intensive care unit, *IQR* interquartile range, *N/A* not available, *NMES* neuromuscular electro-stimulation

^a^Three-arm trial

^b^Information retrieved via personal communication with authors

### Risk of bias and certainty of evidence

**Fig. 2:**
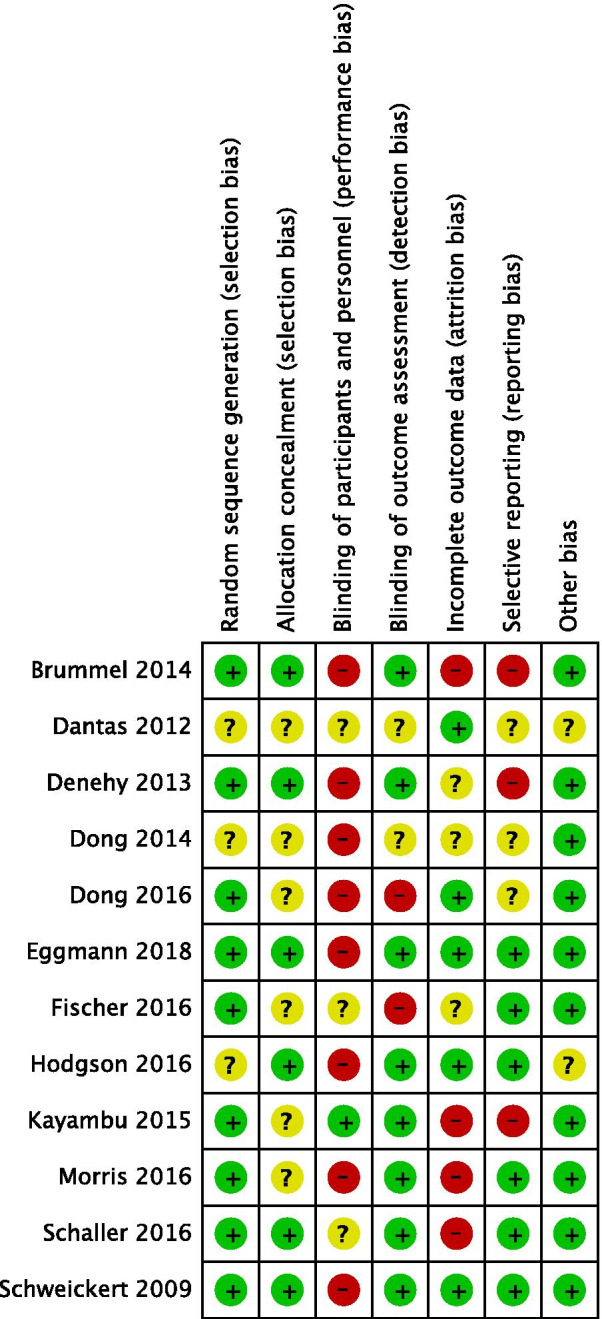
Risk of bias assessment for the included studies

We considered nine out of twelve RCTs to be at high risk of bias in one or more criteria and therefore rated them as of 'poor overall quality' [24–32]. Two studies were judged to be of 'good overall quality' [23, 34] and one study of 'fair overall quality' [33]. The most frequent issues apart from the blinding of participants and personnel were incomplete outcome data and concerns related to selective reporting. Figure 2 shows an overview of the risk of bias assessment (see Additional file 2 for details). While the number of RCTs reporting results for each priority outcome was low, we found no indication for a small study effect that may have influenced our results. The GRADE assessment of the certainty of evidence is presented in Table 3.

**Table 3:** Summary of findings and GRADE assessment for priority outcomes

Outcomes	Anticipated absolute effects (95% CI)		Relative effect (95% CI)	No. of participants (studies)	Certainty of the evidence (GRADE)	Comments
	Late mobilization or standard early mobilization	Systematic early mobilization				
MRC Sum Score (MRC-SS), measured at ICU discharge	*(i) Systematic early vs. late mobilization*		–	104 (1 RCT)	⨁⨁◯◯ LOW^a,b^	
	The median MRC-SS in the comparator group was 48 (0 to 58)	The median MRC-SS in the intervention group was 52 (25 to 58)				
	*(ii) Systematic early vs. standard early mobilization*		–	203 (4 RCTs)	⨁◯◯◯ VERY LOW^a,c,d^	In a sensitivity analysis, omitting the study by Dantas et al. due to a high baseline imbalance in MRC-SS resulted in an MRC-SS in the intervention group, which was 2.2 higher (2.5 lower to 6.9 higher). For that result, the certainty of evidence is judged low (no serious inconsistency)
	The mean MRC-SS in the comparator group in studies ranged from 40.3 to 47.3	The mean MRC-SS in the intervention group was 5.8 higher (1.4 lower to 13.0 higher)				
Patients developing ICUAW, measured at hospital discharge	*(i) Systematic early vs. late mobilization*		RR 0.62 (0.38–1.03)	104 (1 RCT)	⨁⨁◯◯ LOW^a,b^	
	49 per 100	31 per 100				
	*(ii) Systematic early vs. standard early mobilization*		RR 0.90 (0.63–1.27)	395 (3 RCTs)	⨁◯◯◯ VERY LOW^c,e^	
	39 per 100	36 per 100				
6-min Walk Test (6MWT), measured at various time points	(ii) Systematic early vs. standard early mobilization*		–	232 (2 RCTs)	⨁⨁◯◯ LOW^a,f,g^	
	The mean 6MWT distance in the comparator group was 246 m in Eggmann et al. and 267 m in Denehy et al. at hospital discharge	The mean 6MWT distance in the intervention group was 223 in Eggmann et al. and 244.2 in Denehy et al. at hospital discharge				
	The mean change in 6MWT from baseline in the comparator group was 184.3 m at 3 months and 219.5 m at 6 months after hospital discharge in Denehy et al	The mean change in 6MWT from baseline in the intervention group was 63.7 m higher (14.2 to 113.2) at 3 months and 72.6 m higher (9.3 to 135.8) at 6 months in Denehy et al				
Time to walking, measured during hospital stay	*(i) Systematic early vs. late mobilization*		–	104 (1 RCT)	⨁⨁◯◯ LOW^a,b^	
	The median time to walking in the comparator group was 7.3 days (4.9 to 9.6)	The median time to walking in the intervention group was 3.8 days (1.9 to 5.8)				
	*(ii) Systematic early vs. standard early mobilization*		–	53 (2 RCTs)	⨁◯◯◯ VERY LOW^a,c,h^	
	The median time to walking in the comparator group was 6 days in Hodgson et al. and 23 days in Eggmann et al	The median time to walking in the intervention group was 6 days in Hodgson et al. and 8 days in Eggmann et al				
Patients returning to independence from assistance, measured at hospital discharge	(i) Systematic early vs. late mobilization*		RR 1.71 (1.11–2.64)	104 (1 RCT)	⨁⨁◯◯ LOW^a,b^	
	35 per 100	59 per 100				
SF-36 Physical Function Domain Score (PFS), measured 6 months after hospital discharge	*(i) Systematic early vs. late mobilization*		–	161 (1 RCT)	⨁◯◯◯ VERY LOW^a,b,c^	
	The mean SF-36 PFS in the comparator group was 43.6	The mean SF-36 PFS in the intervention group was 12.3 higher (3.9 to 20.8)				
	*(ii) Systematic early vs. standard early mobilization*		–	126 (2 RCTs)	⨁◯◯◯ VERY LOW^a,c,d,e^	
	The mean SF-36 PFS in the comparator group in studies ranged from 42.4 to 75.0	The mean SF-36 PFS in the intervention group was 8.1 higher (15.3 lower to 31.4 higher)				
SF-36 Physical Health Component Summary Score (PCS), measured 6 months after hospital discharge	*(i) Systematic early vs. late mobilization*		–	161 (1 RCT)	⨁◯◯◯ VERY LOW^a,b,c^	
	The mean SF-36 PCS in the comparator group was 33.5	The mean SF-36 PCS in the intervention group was 3.4 higher (0.01 higher to 6.8 higher)				
	*(ii) Systematic early vs. standard early mobilization*		–	152 (2 RCTs)	⨁◯◯◯ VERY LOW^c,e^	
	The mean SF-36 PCS in the comparator group in studies ranged from 42.7 to 44.4	The mean SF-36 PCS in the intervention group was 2.4 lower (6.1 lower to 1.3 higher)				

*CI* confidence interval, *GRADE* Grading of Recommendations Assessment, Development, and Evaluation, *ICU* intensive care unit, *ICUAW* ICU-acquired weakness, *MD* mean difference, *MRC-SS* Medical Research Council Sum Score, *PCS* Physical Health Component Summary Score, *PFS* Physical Function Domain Score, *RCT* randomized controlled trial, *RR* risk ratio, *6MWT* 6-min walk test

^*^Information was available only for one comparator group

^a^Downgraded one point due to imprecision (defined as wide confidence intervals including no effect and/or low overall sample size (defined as < 400 participants for continuous outcomes or below optimal information size for dichotomous outcomes))

^b^Downgraded one point due to only one study contributing to outcome

^c^Downgraded one point as majority of studies judged as of overall poor quality regarding risk of bias

^d^Downgraded one point due to presence of substantial unexplained heterogeneity

^e^Downgraded two points due to high imprecision [wide confidence intervals for absolute effects including important harm and low overall sample size (see definition above)]

^f^Not downgraded as we judged the risk of bias of studies contributing data as not relevant for outcome

^g^Downgraded one point due to only one study contributing to outcome (change from baseline deemed most important aspect of outcome)

^h^Downgraded one point due to only one study contributing to outcome [the second study barely contributed data (*n* = 3)]

### MRC Sum Score

**Fig. 3:**
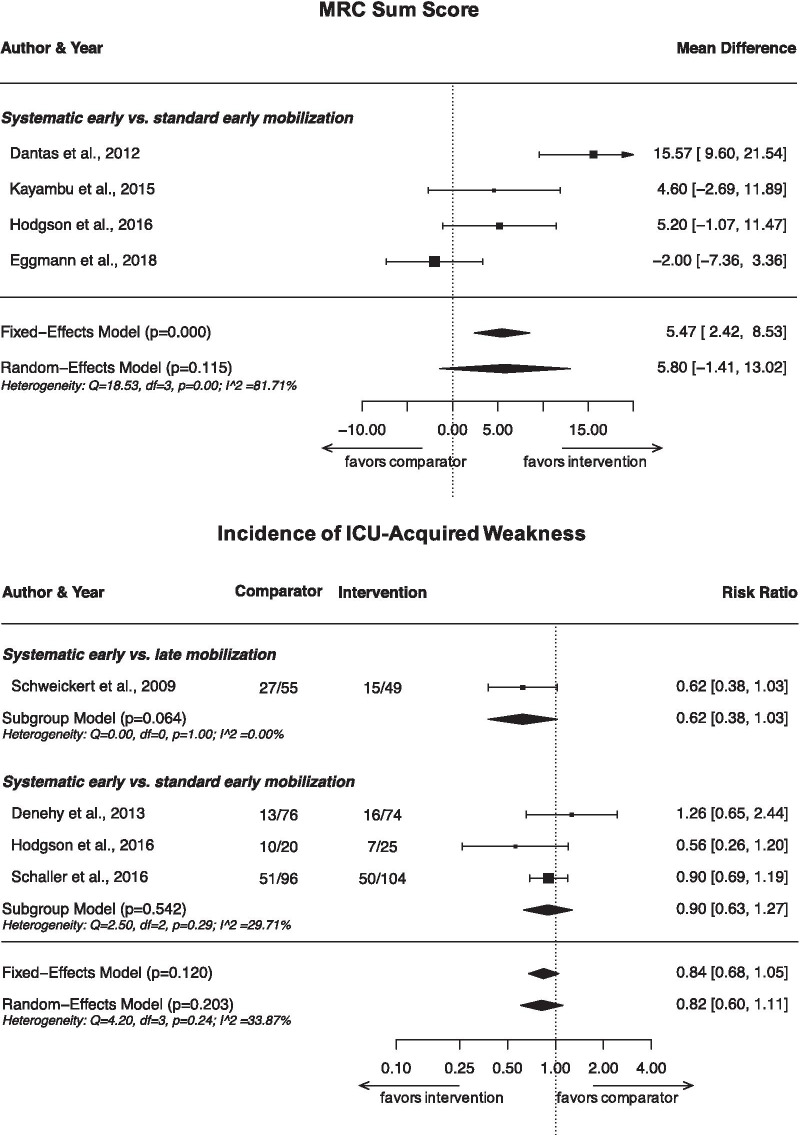
Meta-analysis results on MRC Sum Scores at ICU discharge and proportion of patients developing ICU-acquired weakness during hospitalization

Five studies reported on MRC-SS [23, 24, 28, 31, 34] at ICU discharge. Four studies found no statistically significant difference between systematic early mobilization and late mobilization [23] or standard early mobilization [28, 31, 34]. Dantas et al. reported a statistically significantly higher MRC-SS in favor of systematic early mobilization compared with standard early mobilization [24]. However, the mean MRC-SS of participants in the systematic early mobilization group was already higher at baseline compared to the comparator group. Meta-analysis including data from four studies (203 patients) [24, 28, 31, 34] showed no statistically significant difference in MRC-SS at ICU discharge between systematic early mobilization and standard early mobilization (MD 5.8 points, 95% confidence interval (CI) − 1.4 to 13.0; *p* = 0.12; *I*^2^ = 81.7%; very low certainty; Fig. 3). In a sensitivity analysis, we excluded the study by Dantas et al. due to the baseline imbalance in MRC-SS, which may have affected their results. We found no evidence for a between-group difference in this analysis (MD 2.2; 95% CI − 2.5 to 6.9; *p* = 0.36; *I*^2^ = 41.2%; low certainty).

### Proportion of patients developing ICUAW during hospitalization

While four studies published results on the proportion of patients developing ICUAW during hospitalization [23, 25, 31, 33], none of these found a statistically significantly lower rate of ICUAW in the systematic early mobilization group compared to late mobilization [23] or standard early mobilization groups [25, 31, 33]. A meta-analysis of all four studies (499 patients) showed no statistically significant difference in the incidence of ICUAW between groups (Fig. 3). However, the effects may be clinically meaningful, with a 38% reduction in the risk for developing ICUAW for systematic early mobilization compared with late mobilization (RR 0.62; 95% CI 0.38–1.02; *p* = 0.06; *I*^2^ = 0.0%; one study; low certainty), and a 10% risk reduction for systematic early compared with standard early mobilization (RR 0.90; 95% CI 0.63–1.27; *p* = 0.54; *I*^2^ = 33.3%; very low certainty).

### 6-Min walk test

Only two studies reported results on 6MWT [25, 34], both comparing systematic early with standard early mobilization. 6MWT distances achieved by study participants were comparable between the two studies. Denehy et al. demonstrated an increase in 6MWT distance in both groups from ICU discharge up to 12 months of follow-up [25]. While there was no difference in 6MWT distances between groups beyond ICU discharge, they reported a statistically significantly higher mean change from baseline at 3 months (MD 63.7 m; 95% CI 14.2–113.2; *p* < 0.05) and 12 months (MD 72.7 m; 95% CI 9.3–135.8; *p* < 0.05) in the systematic early mobilization group. Eggmann et al. did not find evidence for a difference in 6MWT distance between groups at hospital discharge [34]. We judged the certainty of evidence for a benefit of systematic early mobilization on 6MWT compared to standard early mobilization as low.

### Time needed until walking

Three studies reported on the time needed by patients until walking for the first time [23, 31, 34]. Schweickert et al. reported a statistically significantly shorter time to walking in the systematic early mobilization group when compared to late mobilization (low certainty) [23]. In contrast, Hodgson et al. did not find a between-group difference when comparing systematic early with standard early mobilization (very low certainty) [31]. Data from Eggmann et al. were insufficient to draw a conclusion [34].

### Proportion of patients returning to independence from assistance

Only the study by Schweickert et al. reported the proportion of patients returning to independence from assistance during hospitalization [23]. They found a statistically significantly higher proportion of patients reaching independence in the systematic early mobilization group compared to the late mobilization group (low certainty).

### SF-36 Physical Function Domain Score

**Fig. 4:**
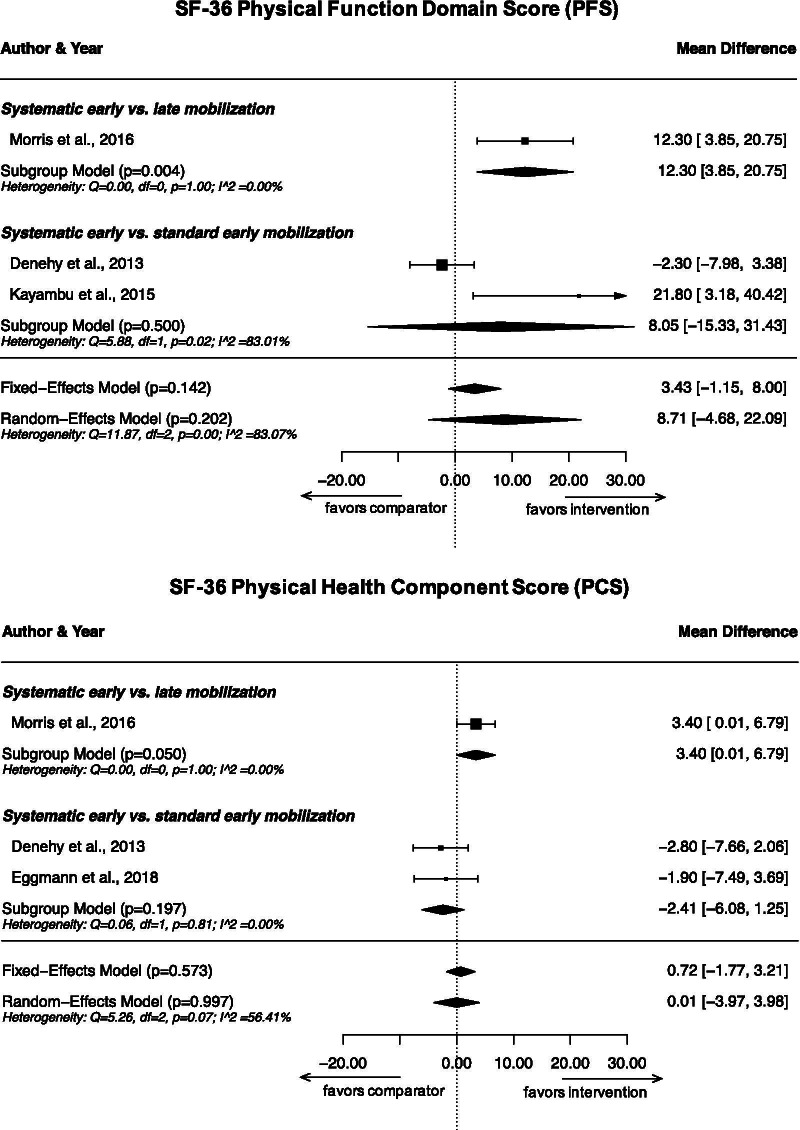
Meta-analysis results on SF-36 Physical Function Domain Scores (PFS) and Physical Health Component Summary Scores (PCS) at 6 months after hospital discharge

Four studies reported results on SF-36 PFS achieved by study participants at 6 months after hospital discharge [25, 28, 32, 34]. There were considerable differences between studies, as Kayambu et al. and Eggmann et al. measured higher scores than Denehy et al. and Morris et al. While Morris et al. found a statistically significant difference between the systematic early mobilization group and the late mobilization group [32], none of the other studies found such a difference compared with standard early mobilization [25, 28, 34]. The results from three studies (287 patients) [25, 28, 32] were included in a meta-analysis (Fig. 4), which showed a statistically significant improvement of SF-36 PFS at 6 months after hospital discharge in the systematic early mobilization group compared to the late mobilization group (MD 12.3; 95% CI 3.9–20.8; *p* = 0.004; one study; very low certainty). However, we found no evidence for such an effect for the comparison of systematic early with standard early mobilization (MD 8.1; 95% CI − 15.3 to 31.4; *p* = 0.50; very low certainty). Heterogeneity for the latter comparison was considerable (*I*^2^ = 83.1%) due to large between-study differences in measured SF-36 PFS [25, 28].

### SF-36 Physical Health Component Summary Score

Out of the three studies reporting results on achieved SF-36 PCS at 6 months after hospital discharge [25, 32, 34], only Morris et al. showed a statistically significant difference between groups [32]. When pooling data from all three studies in a meta-analysis (313 patients), there was some evidence that participants receiving systematic early mobilization achieved higher SF-36 PCS compared to those receiving late mobilization (MD 3.4; 95% CI 0.01–6.8; *p* = 0.050; one study; low certainty). However, there was no evidence for a difference when comparing systematic early with standard early mobilization (MD − 2.4; 95% CI − 6.1 to 1.3; *p* = 0.20; *I*^2^ = 0.0%; low certainty).

### Additional outcomes

In summary, the evidence regarding the benefits of systematic early mobilization was inconclusive across various other outcomes related to muscle strength and physical function (see Additional file 3 for details). While rather weak in general, the evidence was commonly stronger for the comparison between systematic early and late mobilization than for the comparison between systematic early and standard early mobilization. We found no conclusive evidence for an effect on quality of life, cognitive and mental health outcomes, length of ICU or hospital stay, duration of mechanical ventilation or in-hospital or postdischarge mortality. Adverse effects were reported infrequently, with no apparent difference between studies investigating systematic early compared to late mobilization and studies investigating systematic early compared to standard early mobilization.

## Discussion

### Summary of main results

In this systematic review and meta-analysis, we only found little evidence for a beneficial effect of systematic early mobilization on MRC-SS, incidence of ICUAW, 6MWT performance, time needed until walking, proportion of patients returning to independence from assistance, SF-36 PFS and SF-36 PCS. While there was a general trend for an improvement in patient outcomes across trials, we found no evidence in support of systematic early mobilization when compared with standard early mobilization. Effects were generally stronger for the comparison of systematic early with late mobilization, and there was low to very low certainty evidence for a benefit with respect to the time to walking, return to independence, as well as SF-36 PFS and PCS at 6 months after discharge (see Additional file 4). Results were similar between groups for further outcomes related to muscle strength and physical function outcomes, cognitive and mental health outcomes, health-related quality of life, length of stay, duration of ventilation and mortality. Systematic early mobilization appeared safe when conducted under adequate monitoring.

### Interpretation

We found considerable heterogeneity between the included studies. First, there were important differences in study populations. While most studies included a mixed ICU collective, three were limited to postoperative [27, 30] or septic patients [28]. There were large differences between studies in the average length of ICU and hospital stay, as well as in the duration of mechanical ventilation, indicating marked differences in patient recovery between studies. However, on a study level, longer hospitalization and ventilation were barely associated with higher disease severity, as reflected by average Acute Physiologic Assessment and Chronic Health Evaluation (APACHE) scores. We thus consider it likely that these differences were due to variations in standard ICU practices, reasons for ICU admission or other patient characteristics.

Second, there were differences in the interventions provided in the studies. While almost all studies described a diverse set of exercises, tailored to the patient's capability and increasing intensity over time, the systematic early mobilization interventions differed markedly in their scope, intensity and composition between studies. Furthermore, the allocated intervention extended beyond hospital discharge in two studies [25, 26], which did not appear to result in stronger effects on muscle strength or physical function.

Third, the definition of ‘early mobilization’ and its distinction from 'standard care' were often unclear and varied strongly between studies. Standard care was often poorly described, and not all studies reported on differences in the timing of the first mobilization between study arms. Our approach of categorizing studies in comparing systematic early mobilization with either late mobilization or standard early mobilization partially accounted for this issue and revealed that the timing of mobilization in the comparator group may be an important explanatory factor for differences in effects between trials. It is possible that standard practice has evolved after earlier studies on early mobilization found strong effects compared with late mobilization, such as the one by Schweickert et al. [23]. This may have resulted in smaller differences in effects between intervention and control groups, especially in more recent trials which we categorized as comparing systematic early with standard early mobilization.

Finally, we judged most studies to be at risk of bias, which also affected our confidence in most estimates in the GRADE assessment. However, considering results from studies at low risk of bias only would have led to the same conclusions.

### Results in context

Several systematic reviews have addressed early mobilization in recent years [12–15, 35, 36]. Conclusions drawn by these reviews differ as to whether or not the evidence is sufficiently strong to conclude that early mobilization provides a benefit on muscle strength, physical function, quality of life, mortality, length of stay and other outcomes. While Doiron et al. and Castro-Avila et al. reported no statistically significant effects on outcomes related to muscle strength and physical function [14, 15], Fuke et al., Okada et al. and Zang et al. found a statistically significant benefit with early mobilization [12, 13, 35]. This discrepancy primarily stems from slight differences in the inclusion of RCTs. Our review excluded some studies that were included in other reviews due to ineligibility of the study population [37–39] or late initiation of the mobilization intervention [40]. Conversely, we included the recent study by Eggmann et al. [34], which found no evidence for a benefit comparing early mobilization with standard care with a very small timing difference between groups (median 47 vs. 52 h). This may explain why our review did not find sufficient evidence to conclude an effect of early mobilization on muscle strength or physical function outcomes.

As discussed by other authors, the definition of 'early mobilization' varies strongly across studies. [4, 41]. While there is no uniform consensus, the field appears to increasingly define early mobilization as starting within 72 h of ICU admission [4, 6]. Ding et al. attempted to identify the optimal starting time for early mobilization in a network meta-analysis of 15 RCTs, from which they concluded that initiation of mobilization within 72–96 h of mechanical ventilation would be most beneficial for the improvement of ICUAW [36]. Unfortunately, their analysis did not account for timing differences between intervention and comparator groups.

However, the difference in timing between intervention and comparator group may be an important determinant for identifying a benefit of systematic early mobilization in studies. Our findings suggest that systematic early mobilization may be effective when compared to late mobilization, but there was insufficient evidence for a benefit of systematic early mobilization compared to standard early mobilization. Thus, the difference in timing between intervention and comparator groups may be at least as important as the absolute timing of the first mobilization in the intervention group. We consider the separate analysis of different comparator categories a unique strength of our systematic review, as this contrast sheds light on an important issue when interpreting the available evidence on early mobilization. Treatment recommendations on early mobilization need to consider comparator group interventions in trials to judge whether more systematic or earlier mobilization approaches may provide additional clinical benefits over standard care and are cost-effective in the respective context.

### Limitations

Several limitations should be considered when interpreting our findings. First, we defined 'early mobilization' as mobilization starting within 7 days of ICU admission in line with previous reviews on the subject [12, 14]. Using a stricter definition limiting the intervention to early mobilization within 72 h after ICU admission would have led to the exclusion of one study [25] and re-categorization of two studies into the late mobilization category [26, 31]. This would have resulted in an even weaker evidence base for systematic early mobilization compared to both late mobilization and standard early mobilization. However, it would not have altered our main conclusions.

Second, we categorized studies into comparator categories based on predefined criteria. Due to the heterogeneity of 'early mobilization' between studies, it could occur that the comparator in one study was similar in timing or nature to the experimental intervention in another study, or vice versa. This was especially the case in the *standard early mobilization* category. Alternative assessments showed that if studies for which the timing difference between intervention and comparator group was unclear were excluded from analysis, this would not have altered our conclusions for any of the priority outcomes. Had these studies been assigned to the *late mobilization* category, we would have found smaller and not statistically significant effects on MRC-SS, incidence of ICUAW, SF-36 PFS and PCS for the comparison between systematic early and late mobilization. However, our conclusions regarding the comparison between systematic early and standard early mobilization would have remained unchanged. Since our categorization may not fully reflect the timing differences between studies, a more detailed consideration of interventions and comparators in the individual studies may be warranted when making recommendations for practice.

Third, we excluded studies with relevant proportions of neurological, burns, transplant or postoperative patients requiring only short ventilation. This, as well as the language restriction, may have led to the exclusion of some studies that would have provided additional evidence and could have altered our results.

Fourth, we did not conduct a more detailed analysis of the frequency, duration and intensity, or exact implementation of the interventions. While these factors are likely to influence the effectiveness of interventions, the available information did not provide a sufficient basis for such comparisons.

Finally, we did not perform subgroup analyses other than by comparator category. While it is possible that specific patient subgroups may benefit more strongly from early mobilization than others, the available data were insufficient to conduct such subgroup analyses.

## Conclusion

This systematic review and meta-analysis found a beneficial effect of systematic early mobilization in mechanically ventilated adult ICU patients on muscle strength and physical function when compared to late mobilization, but did not find evidence for such an effect when compared to standard early mobilization initiated within 7 days of ICU admission. This contrast widens the perspective on early mobilization in the ICU, highlighting the need to consider the characteristics of comparator interventions when interpreting RCT-based evidence to make recommendations for clinical practice.

## Supplementary Information

**Additional file 1.** PICO, Search strategy, List of excluded studies.

**Additional file 2.** Risk of Bias assessment details.

**Additional file 3.** Full results.

**Additional file 4.** GRADE evidence profile.

## Data Availability

All data generated and/or analyzed during the current study are included within the published article and its additional files.

## Abbreviations

AMSTAR: Assessing the Methodology of Systematic Reviews
APACHE: Acute Physiologic Assessment and Chronic Health Evaluation
CI: Confidence interval
GRADE: Grading of Recommendations Assessment, Development and Evaluation
ICU: Intensive care unit
ICUAW: Intensive care unit-acquired weakness
IQR: Interquartile range
MD: Mean difference
MRC-SS: Medical Research Council Sum Score
PRISMA: Preferred Reporting Items for Systematic Reviews and Meta-Analyses
RCT: Randomized controlled trial
RR: Risk ratio
SF-36 PFS: Short Form 36 Physical Function Domain Score
SF-36 PCS: Short Form 36 Physical Health Component Summary Score
6MWT: 6-Min Walk Test
