# Supplementary material for: Systematic early versus late mobilization or standard early mobilization in mechanically ventilated adult ICU patients: systematic review and meta-analysis
Source: Crit Care. 2021 Jan 6;25:16. doi: 10.1186/s13054-020-03446-9 (PMC7789482; doi:10.1186/s13054-020-03446-9)
Supplement: Supplementary file 1 — Additional file 1. PICO, Search strategy, List of excluded studies. [file 13054_2020_3446_MOESM1_ESM.docx]

**Additional File 1**

**Eligibility Criteria (PICO)**

Population

Adult ICU patients (≥18 years) requiring ventilation support (i.e., invasive or non-invasive mechanical ventilation)

Intervention

Systematic early mobilization: Mobilization activities initiated within 7 days after ICU admission and performed according to a clearly defined protocol or specific clinical criteria in all patients without contraindications.

Comparators

i. *Late mobilization*: Mobilization activities initiated 7 days or more after ICU admission (also considered to be less systematic in general).

ii. *Standard early mobilization*: Mobilization activities initiated within 7 days after ICU admission, but less systematically (i.e., in a less protocol-driven and consistent manner, according to less strict criteria or not in all patients without contraindications).

iii. *No mobilization*: No actual mobilization measures provided (sham intervention or no intervention).

Outcomes

*Muscle strength*: Medical Research Council Sum Score (MRC-SS)*, incidence of ICU-acquired weakness (ICUAW)*, hand-held dynamometry, handgrip strength.

*Functional mobility*: Barthel Index, Activities of Daily Living (ADL), Functional Independence Measure (FIM), Physical Function in the ICU Test (PFIT), Timed Up-and-Go test (TUG), 6-minute walk test (6MWT)*, distance walked without assistance, time to first time out of bed, time to standing, time to walking*, proportion of patients returning to independence from assistance*, SF-36 Physical Function Domain Score (PFS)*, SF-36 Physical Health Component Summary Score (PCS)*.

*Quality of life*: Health-related quality of life at ICU discharge, hospital discharge, 6 months and 12 months (e.g. EQ-5D, SF-36 overall score).

*Cognitive function and mental health*: Any instruments (e.g. Mini-Mental State Exam (MMSE), Hospital Anxiety and Depression Score (HADS), SF-36 Mental Health Domain Score (MHS) and SF-36 Mental Health Component Summary Score (MCS)), delirium duration, delirium-free days.

*Mortality*: ICU mortality, in-hospital mortality, mortality at 3 months, mortality at 6 months, mortality at 12 months.

*Additional outcomes*: Duration of mechanical ventilation, ventilator-free days, ICU length of stay, hospital length of stay, duration of rehabilitation after discharge^1^, time to return to work^1^.

*Safety outcomes*: Accidents and fractures (during mobilization activities, outside mobilization activities), dislodging of catheters, loss of muscle tone, hypotension, pain due to insertion and reinsertion of catheters.

** prioritized by ICU Experts as most clinically relevant priority outcomes.*

*^1^ no data found in systematic review.*

Time points

ICU discharge, hospital discharge, 3 months after hospital discharge, 6 months after hospital discharge, 12 months after hospital discharge.

**Search Strategies**

Search strategy for systematic reviews (04 Dec 2018)

*Medline (Pubmed) strategy*

("intensive care"[tiab] OR "ICU"[tiab] OR "critical care"[tiab] OR "critically ill"[tiab] OR "mechanical ventilation"[tiab] OR "ventilation support"[tiab] OR "PICS"[tiab] OR "ICUAW"[tiab]) AND ("exercise"[tiab] OR "rehabilitation"[tiab] OR "physical therapy"[tiab] OR "physiotherapy"[tiab] OR "mobilisation"[tiab] OR "mobilization"[tiab] OR "early mobility"[tiab] OR "physical fitness"[tiab] OR "muscle training"[tiab] OR "diary"[tiab]) AND (Meta-Analysis[ptyp] OR systematic[sb] OR "meta-analysis"[tiab] OR "systematic review"[tiab]) AND ("2015/01/01"[PDAT] : "2018/12/04"[PDAT])

*Cochrane Reviews (The Cochrane Library) strategy*

("intensive care":ti,ab OR ICU:ti,ab OR "critical care":ti,ab OR "critically ill":ti,ab OR "mechanical ventilation":ti,ab OR "ventilation support":ti,ab OR PICS:ti,ab OR ICUAW:ti,ab) AND (exercise:ti,ab OR rehabilitation:ti,ab OR "physical therapy":ti,ab OR physiotherapy:ti,ab OR mobilisation:ti,ab OR mobilization:ti,ab OR "early mobility":ti,ab OR "physical fitness":ti,ab OR "muscle training":ti,ab OR diary:ti,ab) AND (meta-analysis:ti,ab OR "systematic review":ti,ab)

*Filter:* publications from 01/01/2015 to 04/12/2018

Search Strategies for Follow-Up Searches (17 Jan 2019)

*Medline (Ovid) strategy based on Doiron et al. 2018*

1 exp Intensive Care Units/ or Critical Illness/ or exp Critical Care/ or (critical* adj3 (ill* or care*)).tw. or intensive care.tw. or (icu or icuaw).tw.

2 exp Exercise Therapy/ or exp Physical Therapy Modalities/ or Occupational Therapy/ or (mobilizat* or mobilisat* or mobility).tw. or exercis*.tw. or (therap* adj3 (physical or exercise or occupation*)).tw. or ((bed or daily living) adj3 activit*).tw. or (training or pregait or pre-gait or walk* or adl or physiotherap* or ambulation).tw. or ((cycle or bicycle) adj1 ergomet*).tw.

3 ((randomized controlled trial or controlled clinical trial).pt. or clinical trial.sh. or (randomized or randomised or randomly).ti,ab. or trial.ti. or placebo.ti,ab.) not (animals not humans).sh.

4 1 and 2 and 3

5 4 and (201706* or 201707* or 201708* or 201709* or 201710* or 201711* or 201712* or 2018* or 2019*).dp,ed,ep,ez.

*Medline (Ovid) strategy based on Fuke et al. 2018*

1 ("critical ill" or "critical illness" or "critical care" or "intensive care" or "mechanical ventilation" or "mechanical ventilated" or "postoperative care").mp.

2 (rehabilitation or "physical therapy" or physiotherapy or exercise or mobilization or "mobility intervention" or "muscle training").mp.

3 ("Activities of Daily Living" or "Quality of Life" or "postintensive care syndrome" or "motor function" or "Physical Functioning" or "functional status" or "physical function" or "ventilator days" or "quality of life" or (walking or walk) or muscle or polyneuromyopathy or "length of stay" or "length of ICU stay" or "length of hospital stay" or "intubation period" or "duration of mechanical ventilation" or re-admission or "functional outcome" or "ICU-acquired weakness" or "ICU-acquired paresis" or ICUAW or "ICUAW" or "intensive care unit acquired weakness" or "critical illness polyneuropathy" or "critical illness myopathy" or "critical illness neuromyopathy" or "acute quadriplegic myopathy" or "thick filament myopathy" or "acute necrotizing myopathy of intensive care" or "acute corticosteroid myopathy" or "critical illness neuromuscula syndromes" or "Tower test" or "Timed Up and Go Test" or "dysexecutive questionnaire" or FAQ or "EQ-5D VAS" or 6MWD or "6-min walking distance" or "Quadriceps force, and self-perceived functional status" or "SF-36 PF" or MRC or "Medical Research Council" or "AQoL utility" or "EQ-5D" or PFIT or "physical functional ICU test" or "Hospital Anxiety and Depression Scale" or "Hand-grip strength").mp.

4 ((randomized controlled trial or controlled clinical trial).pt. or clinical trial.sh. or (randomized or randomised or randomly).ti,ab. or trial.ti. or placebo.ti,ab.)) not (animals not humans).sh.

5 1 and 2 and 3 and 4

6 5 and (201604* or 201605* or 201606* or 201607* or 201608* or 201609* or 201610* or 201611* or

*Medline (Ovid) strategy based on Castro-Avila et al. 2015*

1 Critical Care/ or critical care.mp. or intensive care units/ or intensive care unit?.mp. or burn units/ or burn unit?.mp. or coronary care units/ or coronary care unit?.mp. or recovery room/ or recovery room?.mp. or respiratory care units/ or respiratory care unit?.mp. or Critical Illness/rh or Critical Illness/ or (critical illness or critically ill).mp. or *Intensive Care/ or intensive care.mp. or intensive treatment unit?.mp. or intensive therapy unit?.mp. or high dependency unit?.mp. or ICU.mp. or HDU.mp.

2 exp Rehabilitation/ or rehabilitat*.mp. or exp Physical Therapy Modalities/ or physical therapy modalit?.mp. or physical therap*.mp. or physiotherap*.mp. or kinesiotherap*.mp. or exp Exercise Therapy/ or exercise therap*.mp. or physical exertion/ or physical exertion.mp. or Early Ambulation/ or Early Ambulation.mp. or mobilization.mp. or mobilisation.mp. or Muscle Weakness/rh or Muscle Weakness/th or Neuromuscular Diseases/rh

3 ((randomized controlled trial or controlled clinical trial).pt. or clinical trial.sh. or (randomized or randomised or randomly).ti,ab. or trial.ti. or placebo.ti,ab.) not (animals not humans).sh.

4 1 and 2 and 3

5 4 and (201402* or 201403* or 201404* or 201405* or 201406* or 201407* or 201408* or 201409* or 201410* or 201411* or 201412* or 2015* or 2016* or 2017* or 2018* or 2019*).dp,ed,ep,ez.

*Embase (Embase.com/Elsevier) strategy based on Doiron et al. 2018*

#1 icu:ab,ti OR icuaw:ab,ti OR 'intensive care':ab,ti OR ((critical* NEAR/3 (ill* OR care)):ab,ti) OR 'intensive care'/exp OR 'critical illness'/de OR 'intensive care unit'/de

#2 training:ab,ti OR pregait:ab,ti OR 'pre-gait':ab,ti OR walk*:ab,ti OR adl:ab,ti OR physiotherapy*:ab,ti OR (((cycle OR bicycle) NEAR/1 ergomet*):ab,ti) OR ambulation:ab,ti OR (((bed OR 'daily living') NEAR/3 activity):ab,ti) OR ((therap* NEAR/3 (physical* OR exercise OR occupation*)):ab,ti) OR exercis*:ab,ti OR mobiliz*:ab,ti OR mobilis*:ab,ti OR mobility:ab,ti OR 'occupational therapy'/de OR 'physiotherapy'/exp OR 'kinesiotherapy'/exp

#3 ('controlled clinical trial'/exp OR randomized:ti,ab OR randomised:ti,ab OR randomly:ti,ab OR trial:ti OR placebo:ti,ab) NOT ([animals]/lim NOT [humans]/lim)

#4 #1 AND #2 AND #3

#5 #4 AND [1-6-2017]/sd NOT [conference abstract]/lim

*Embase (Embase.com/Elsevier) strategy based on Fuke et al. 2018*

#1 'critical ill':ab,ti OR 'critical illness':ab,ti OR 'critical care':ab,ti OR 'intensive care':ab,ti OR 'mechanical ventilation':ab,ti OR 'mechanical ventilated':ab,ti OR 'postoperative care':ab,ti

#2 rehabilitation:ab,ti OR 'physical therapy':ab,ti OR physiotherapy:ab,ti OR exercise:ab,ti OR mobilization:ab,ti OR 'mobility intervention':ab,ti OR 'muscle training':ab,ti

#3 'activities of daily living':ab,ti OR 'post-intensive care syndrome':ab,ti OR 'motor function':ab,ti OR 'physical functioning':ab,ti OR 'functional status':ab,ti OR 'physical function':ab,ti OR 'ventilator days':ab,ti OR 'quality of life':ab,ti OR walking:ab,ti OR walk:ab,ti OR muscle:ab,ti OR polyneuromyopathy:ab,ti OR 'length of stay':ab,ti OR 'length of icu stay':ab,ti OR 'length of hospital stay':ab,ti OR 'intubation period':ab,ti OR 'duration of mechanical ventilation':ab,ti OR 'readmission': ab,ti OR 'functional outcome':ab,ti OR 'icu-acquired weakness':ab,ti OR 'icu-acquired paresis':ab,ti OR icuaw:ab,ti OR 'icu-aw':ab,ti OR 'intensive care unit acquired weakness':ab,ti OR 'critical illness polyneuropathy':ab,ti OR 'critical illness myopathy':ab,ti OR 'critical illness neuromyopathy':ab,ti OR 'acute quadriplegic myopathy':ab,ti OR 'thick filament myopathy':ab,ti OR 'acute necrotizing myopathy of intensive care':ab,ti OR 'acute corticosteroid myopathy':ab,ti OR 'critical illness neuromuscula syndromes':ab,ti OR 'tower test':ab,ti OR 'timed up and go test':ab,ti OR 'dysexecutive questionnaire':ab,ti OR faq:ab,ti OR 'eq-5d vas':ab,ti OR 6mwd:ab,ti OR '6-min walking distance':ab,ti OR 'quadriceps force, and self-perceived functional status':ab,ti OR 'sf-36 pf':ab,ti OR mrc:ab,ti OR 'medical research council':ab,ti OR 'aqol utility':ab,ti OR 'eq-5d':ab,ti OR pfit:ab,ti OR 'physical functional icu test':ab,ti OR 'hospital anxiety and depression scale':ab,ti OR 'hand-grip strength':ab,ti

#4 ('controlled clinical trial'/exp OR randomized:ti,ab OR randomised:ti,ab OR randomly:ti,ab OR trial:ti OR placebo:ti,ab) NOT ([animals]/lim NOT [humans]/lim)

#5 #1 AND #2 AND #3 AND #4

#6 #5 AND [1-4-2017]/sd NOT [conference abstract]/lim

*Embase (Embase.com/Elsevier) strategy based on Castro-Avila et al. 2015*

#1 'intensive care'/de OR 'critical care':de,lnk,ab,ti OR 'intensive care unit'/de OR 'intensive care unit*':de,lnk,ab,ti OR 'burn unit'/de OR 'burn unit*':de,lnk,ab,ti OR 'coronary care unit'/de OR 'coronary care unit*':de,lnk,ab,ti OR 'recovery room'/de OR 'recovery room*':de,lnk,ab,ti OR 'respiratory care unit*':de,lnk,ab,ti OR 'critical illness'/de OR 'critical illness':de,lnk,ab,ti OR 'critically ill':de,lnk,ab,ti OR 'intensive care'/mj OR 'intensive care':de,lnk,ab,ti OR 'intensive treatment unit*':de,lnk,ab,ti OR 'intensive therapy unit*':de,lnk,ab,ti OR 'high dependency unit*':de,lnk,ab,ti OR icu:de,lnk,ab,ti OR hdu:de,lnk,ab,ti

#2 'rehabilitation'/exp OR rehabilitat*:de,lnk,ab,ti OR 'physiotherapy'/exp OR 'physical therapy modalit*':de,lnk,ab,ti OR 'physical therap*':de,lnk,ab,ti OR physiotherap*:de,lnk,ab,ti OR kinesiotherap*:de,lnk,ab,ti OR 'kinesiotherapy'/exp OR 'exercise therap*':de,lnk,ab,ti OR 'exercise'/de OR 'physical exertion':de,lnk,ab,ti OR 'mobilization'/de OR 'early ambulation':de,lnk,ab,ti OR mobilization:de,lnk,ab,ti OR mobilisation:de,lnk,ab,ti OR 'muscle weakness'/dm_rh,dm_th OR 'neuromuscular disease'/dm_rh

#3 ('controlled clinical trial'/exp OR randomized:ti,ab OR randomised:ti,ab OR randomly:ti,ab OR trial:ti OR placebo:ti,ab) NOT ([animals]/lim NOT [humans]/lim)

#4 #1 AND #2 AND #3

#5 #4 AND [1-2-2014]/sd NOT [conference abstract]/lim

*CINAHL (EBSCOhost) strategy based on Doiron et al. 2018*

(TI ((cycle or bicycle) N1 ergomet*) OR AB ((cycle or bicycle) N1 ergomet*) OR TI (training or pregait or pre-gait or walk* or adl or physiotherap* or ambulation) OR AB (training or pregait or pregait or walk* or adl or physiotherap* or ambulation) TI ((bed or daily living) N3 activit*) OR AB ((bed or daily living) N3 activit*) TI (therap* N3 (physical or exercise or occupation*)) OR AB (therap* N3 (physical or exercise or occupation*)) TI exercis* OR AB exercis* TI (mobilizat* or mobilisat* or mobility) OR AB (mobilizat* or mobilisat* or mobility) (MH "Occupational Therapy+") (MH "Physical Therapy+") (MH "Therapeutic Exercise+") ) AND ( TI (icu or icuaw) OR AB (icu or icuaw) OR TI intensive careOR AB intensive care OR TI (critical*N3 (ill* or care*)) OR AB (critical* N3 (ill* or care*)) OR (MH "Critical Care") OR (MH "Critical Illness") OR (MH "Intensive Care Units+") ) AND ( (MH "Randomized Controlled Trials+") OR TI (randomized or randomised or randomly or trial or placebo) OR AB (randomized or randomised or randomly or placebo) NOT ((MH "Animals+") NOT (MH "Human")))

*Limiters*: Published Date: 20170601; Search modes: Find all my search terms

*CINAHL (EBSCOhost) strategy based on Fuke et al. 2018*

(("critical ill" OR "critical illness" OR "critical care" OR "intensive care" OR "mechanical ventilation" OR "mechanical ventilated" OR "postoperative care") ) AND ( (rehabilitation OR "physical therapy" OR physiotherapy OR exercise OR mobilization OR "mobility intervention" OR "muscle training") ) AND ( ("Activities of Daily Living" OR "Quality of Life" OR "post-intensive care syndrome" OR "motor function" OR "Physical Functioning" OR "functional status" OR "physical function" OR "ventilator days" OR "quality of life" OR (walking OR walk) OR muscle OR polyneuromyopathy OR "length of stay" OR "length of ICU stay" OR "length of hospital stay" OR "intubation period" OR "duration of mechanical ventilation" OR re-admission OR "functional outcome" OR "ICU-acquired weakness" OR "ICU-acquired paresis" OR ICUAW OR "ICU-AW" OR "intensive care unit acquired weakness" OR "critical illness polyneuropathy" OR "critical illness myopathy" OR "critical illness neuromyopathy" OR "acute quadriplegic myopathy" OR "thick filament myopathy" OR "acute necrotizing myopathy of intensive care" OR "acute corticosteroid myopathy" OR "critical illness neuromuscula syndromes" OR "Tower test" OR "Timed Up and Go Test" OR "dysexecutive questionnaire" OR FAQ OR "EQ-5D VAS" OR 6MWD OR "6-min walking distance" OR "Quadriceps force, and self-perceived functional status" OR "SF-36 PF" OR MRC OR "Medical Research Council" OR "AQoL utility" OR "EQ-5D" OR PFIT OR "physical functional ICU test" OR "Hospital Anxiety and Depression Scale" OR "Hand-grip strength") ) AND ( (MH "Randomized Controlled Trials+") OR TI (randomized or randomised or randomly or trial or placebo) OR AB (randomized or randomised or randomly or placebo) NOT ((MH "Animals+") NOT (MH "Human")))

*Limiters*: Published Date: 20160401; Search modes: Find all my search terms

*CINAHL (EBSCOhost) strategy based on Castro-Avila et al. 2015*

((MH "Critical Care") OR "critical care" OR (MH "Intensive Care Units") OR "intensive care unit? " OR (MH "Burn Units") OR "burn unit? " OR (MH "Coronary Care Units") OR "coronary care unit?" OR (MH "Post Anesthesia Care Units") OR "recovery room?" OR (MH "Respiratory Care Units") OR "respiratory care unit?" OR (MH "Critical Illness") OR (MH "Critical Illness/RH") OR "critical illness" OR "critically ill" OR "intensive care" OR "intensive treatment unit?" OR "intensive therapy unit?" OR "high dependency unit?" OR ICU OR HDU ) AND ( (MH "Rehabilitation+") OR rehabilitat* OR (MH "Physical Therapy+") OR "physical therapy modalit?" OR "physical therap*" OR physiotherap* OR kinesiotherap* OR (MH "Therapeutic Exercise") OR "exercise therap*" OR "physical exertion" OR (MH "Early Ambulation") OR "Early Ambulation" OR mobilization or mobilisation OR (MH "Muscle Weakness/RH/TH") OR (MH "Neuromuscular Diseases/RH") ) AND ( (MH "Randomized Controlled Trials+") OR TI (randomized or randomised or randomly or trial or placebo) OR AB (randomized or randomised or randomly or placebo) NOT ((MH "Animals+") NOT (MH "Human")))

*Limiters*: Published Date: 20140201; Search modes: Find all my search terms

*CENTRAL (The Cochrane Library) strategy based on Doiron et al. 2018*

#1 ([mh "Intensive Care Units"] OR [mh ^"Critical Illness"] OR [mh "Critical Care"] OR (critical* NEAR3 (ill* OR care*)):ti,ab OR "intensive care":ti,ab OR (icu OR icuaw):ti,ab)

#2 ([mh "Exercise Therapy"] OR [mh "Physical Therapy Modalities"] OR [mh "Occupational Therapy"] OR (mobilizat* OR mobilisat* OR mobility):ti,ab OR exercis*:ti,ab OR (therap* NEAR3 (physical OR exercise OR occupation*)):ti,ab OR ((bed OR "daily living") NEAR3 activit*):ti,ab OR (training OR pregait OR pre-gait OR walk* OR adl OR physiotherap* OR ambulation):ti,ab OR ((cycle OR bicycle) NEAR1 ergomet*):ti,ab)

#3 #1 AND #2

*Limiter*: from June 2017

*CENTRAL (The Cochrane Library) strategy based on Fuke et al. 2018*

#1 ("critical ill" OR "critical illness" OR "critical care" OR "intensive care" OR "mechanical ventilation" OR "mechanical ventilated" OR "postoperative care"):kw,ti,ab

#2 (rehabilitation OR "physical therapy" OR physiotherapy OR exercise OR mobilization OR "mobility intervention" OR "muscle training"):kw,ti,ab

#3 ("Activities of Daily Living" OR "Quality of Life" OR "post-intensive care syndrome" OR "motor function" OR "Physical Functioning" OR "functional status" OR "physical function" OR "ventilator days" OR "quality of life" OR (walking OR walk) OR muscle OR polyneuromyopathy OR "length of stay" OR "length of ICU stay" OR "length of hospital stay" OR "intubation period" OR "duration of mechanical ventilation" OR re-admission OR "functional outcome" OR "ICU-acquired weakness" OR "ICU-acquired paresis" OR ICUAW OR "ICU-AW" OR "intensive care unit acquired weakness" OR "critical illness polyneuropathy" OR "critical illness myopathy" OR "critical illness neuromyopathy" OR "acute quadriplegic myopathy" OR "thick filament myopathy" OR "acute necrotizing myopathy of intensive care" OR "acute corticosteroid myopathy" OR "critical illness neuromuscula syndromes" OR "Tower test" OR "Timed Up and Go Test" OR "dysexecutive questionnaire" OR FAQ OR "EQ-5D VAS" OR 6MWD OR "6-min walking distance" OR "Quadriceps force, and self-perceived functional status" OR "SF-36 PF" OR MRC OR "Medical Research Council" OR "AQoL utility" OR "EQ-5D" OR PFIT OR "physical functional ICU test" OR "Hospital Anxiety and Depression

#4 #1 AND #2 AND #3

*Limiter*: from Apr 2016

*CENTRAL (The Cochrane Library) strategy based on Castro-Avila et al. 2015*

#1 ("critical care" OR "intensive care unit? " OR "burn unit? " OR "coronary care unit?" OR "recovery room?" OR "respiratory care unit?" OR "critical illness" OR "critically ill" OR "intensive care" OR "intensive treatment unit?" OR "intensive therapy unit?" OR "high dependency unit?" OR ICU OR

#2 (rehabilitat* OR "physical therapy modalit?" OR "physical therap*" OR physiotherap* OR kinesiotherap* OR "exercise therap*" OR "physical exertion" OR "Early Ambulation" OR mobilization or mobilisation):kw,ti,ab

#3 #1 AND #2

*Limiter*: from Feb 2014

**List of Excluded Studies, with Reason**

*Abstract only (n=43)*

1. Schweickert W, Poston J, Esbrook C, et al. Temporal Relation of Early Mobilization on Recovery of Functional Independence in Mechanically Ventilated Patients. A92 FIVE RANDOMIZED CLINICAL TRIALS WITH EDITORIAL DISCUSSION 2009;:A2168. doi:10.1164/ajrccm-conference.2009.179.1_MeetingAbstracts.A2168
2. Hanekom S., Louw Q., Coetzee A. Physiotherapy management of critically ill patients guided by an evidence based protocol is safe and effective: A preliminary study. Intensive Care Med 2010;36:S324. doi:10.1007/s00134-010-2000-8
3. Malicdem M.G., Cruz B.O.-D., Punzal P., et al. Outcome of pulmonary rehabilitation among difficult to wean patients admitted at the philippine heart center - A randomized controlled study. Respirology 2010;15:99. doi:10.1111/j.1400-1843.2010.01865.x
4. Patel B, Poston J, Pohlman A, et al. Complications Of Critical Illness In Mechanically Ventilated Patients In A Randomized Controlled Trial Of Early Mobilization. D49 CLINICAL TRIALS IN CRITICAL CARE 2010;:A6033–A6033. doi:10.1164/ajrccm-conference.2010.181.1_MeetingAbstracts.A6033
5. Arikan H, Turan HN, Degirmenci B, et al. Comparison in the efficacy of mobilization and active cycle of breathing technique in coronary artery bypass greft surgery. European Respiratory Journal 2011;38:2979.
6. Berney SC, Haines K, Warrillow S, et al. The Safety And Feasibility Of Exercise Rehabilitation In The ICU. A104 INTENSIVE CARE UNIT ORGANIZATION, OUTCOMES, AND RESEARCH 2011;:A2385–A2385. doi:10.1164/ajrccm-conference.2011.183.1_MeetingAbstracts.A2385
7. Denehy L, Berney S, Skinner E, et al. Evaluation Of Exercise Rehabilitation For Survivors Of Intensive Care: An Assessor Blinded Randomised Controlled Trial. B25 MONITORING AND NON-PULMONARY CRITICAL CARE 2011;:A2642–A2642. doi:10.1164/ajrccm-conference.2011.183.1_MeetingAbstracts.A2642
8. Evans J, Tsekouras C, Johnson K, et al. Effect of Early Mobilization Efforts on Postoperative Length of Stay After Cardiac Transplant and Left Ventricular Assist Device Surgery. Critical Care Nurse 2011;31:e50-1.
9. Gerovasili V., Karatzanos L., Zervakis D., et al. Electrical muscle stimulation is an effective form of exercise and early mobilization in ICU patients. Am J Respir Crit Care Med 2011;183.http://www.embase.com/search/results?subaction=viewrecord&from=export&id=L70848092
10. Ali MS, Talwar D, Singh RK, et al. Controlled Trial Of Short Term (3 Weeks) Pulmonary Rehabilitation In COPD Following Acute Exacerbation. B45 EXACERBATIONS OF COPD: PREVENTION, TREATMENT AND OUTCOMES 2012;:A3034–A3034. doi:10.1164/ajrccm-conference.2012.185.1_MeetingAbstracts.A3034
11. Brummel NE, Jackson JC, Girard TD, et al. Feasibility Of An Early Physical And Cognitive Rehabilitation Protocol For Critically Ill Patients: The Activity And Cognitive Therapy In The ICU (ACT-ICU) Trial. C14 CLINICAL TRIALS IN CRITICAL CARE 2012;:A3885–A3885. doi:10.1164/ajrccm-conference.2012.185.1_MeetingAbstracts.A3885
12. Paternostro-Sluga T, Gruther W. Intensive Physical Therapy Reduces Length of Hospital Stay in Critically Ill Patients. PM&R 2012;4:S310–1. doi:10.1016/j.pmrj.2012.09.965
13. Paternostro-Sluga T, Hiesmayr M, Janda D, et al. Early Neuromuscular Electrical Stimulation for Intensive Care Unit Patients: Effect on Muscle Strength and Urinary Nitrogen Excretion. PM&R 2012;4:S310. doi:10.1016/j.pmrj.2012.09.964
14. Beros J, Khadka G, Duffner L, et al. Does Neuromuscular Electric Stimulation Of The Quadriceps Affect Mobility In Patients Weaning From Prolonged Ventilation? B104 ICU WEAKNESS ON THE RUN: EXERCISE, ELECTRICAL STIMULATION, AND PHARMACOTHERAPY 2013;:A3618–A3618. doi:10.1164/ajrccm-conference.2013.187.1_MeetingAbstracts.A3618
15. Files D, Morris P, Shrestha S, et al. Randomized, controlled pilot study of early rehabilitation strategies in acute respiratory failure. Critical Care 2013;17:P540. doi:10.1186/cc12478
16. Goodman J, Walker W, Wright J, et al. Project PIX (Post Intensive care eXercise): impact on physical fitness and focus group analysis of quality of life following exercise rehabilitation. Crit Care 2013;17:P534. doi:10.1186/cc12472
17. Kho ME, Martin RA, Toonstra AL, et al. Le Tour De ICU: Feasibility And Safety Of Routine Use Of In-Bed Cycling For Physical Rehabilitation In The Intensive Care Unit (ICU). B104 ICU WEAKNESS ON THE RUN: EXERCISE, ELECTRICAL STIMULATION, AND PHARMACOTHERAPY 2013;:A3620–A3620. doi:10.1164/ajrccm-conference.2013.187.1_MeetingAbstracts.A3620
18. Wolfe KS, Wendlandt BN, Patel SB, et al. Long-Term Survival And Health Care Utilization Of Mechanically Ventilated Patients In A Randomized Controlled Trial Of Early Mobilization. D16 RANDOMIZED AND OBSERVATIONAL STUDIES IN CRITICAL CARE 2013;:A5235–A5235. doi:10.1164/ajrccm-conference.2013.187.1_MeetingAbstracts.A5235
19. Emerson K, Hu BB, Smith C, et al. Impact Of A Collaborative Multidisciplinary Team On ICU Delirium. Critical Care Medicine 2014;42:A1501–2.
20. Kho ME, Truong AD, Zanni JM, et al. Neuromuscular Electrical Stimulation (NMES) In Mechanically Ventilated Patients: A Randomized, Sham-Controlled Pilot Trial With Blinded Outcome Assessment. B109 ICU ACQUIRED MUSCLE WEAKNESS: MAKING PROGRESS? 2014;:A3881–A3881. doi:10.1164/ajrccm-conference.2014.189.1_MeetingAbstracts.A3881
21. Eggmann S, Verra ML, Luder G, et al. Physiological effects and safety of an early, combined endurance and resistance training in mechanically ventilated, critically ill patients. Physiotherapy 2015;101:e344–5. doi:10.1016/j.physio.2015.03.553
22. Fares S, Laghi F, Duffner LA, et al. Impact of Neuromuscular Electrical Stimulation on Quadriceps Size and Functional Activity in Patients Weaning from Prolonged Ventilation. A104 MOVING THE NEEDLE ON ICU-ASSOCIATED NEUROMUSCULAR WEAKNESS 2015;:A2294–A2294. doi:10.1164/ajrccm-conference.2015.191.1_MeetingAbstracts.A2294
23. Goll M, Wollersheim T, Haas K, et al. Randomised controlled trial using daily electrical muscle stimulation (EMS) in critically ill patients to prevent intensive care unit (icu) acquired weakness (ICUAW). Intensive Care Med Exp 2015;3. doi:10.1186/2197-425X-3-S1-A809
24. Hadjibalassi M, Lambrinou E, Papastavrou E, et al. Effects of a psycho-cognitive nursing intervention on critical care patients: pain and anxiety levels. CONNECT: The World of Critical Care Nursing 2015;9:158–158.
25. Kayambu G, Boots R, Paratz J. Early physical rehabilitation in intensive care patients with sepsis syndromes—a randomised controlled trial. Physiotherapy 2015;101:e735. doi:10.1016/j.physio.2015.03.3597
26. Santos L, Lemos F, Bianchi T, et al. Early ambulation using a cycle ergometer on quadriceps muscle morphology in mechanically ventilated critically ill patients in the intensive care unit: a randomized controlled trial. Intensive Care Med Exp 2015;3. doi:10.1186/2197-425X-3-S1-A551
27. Bissett B, Leditschke IA, Neeman T, et al. Inspiratory Muscle Training to Enhance Recovery from Prolonged Mechanical Ventilation: A Randomized Trial. A95 CRITICAL CARE: RECOVERY OF PHYSICAL FUNCTION AFTER CRITICAL ILLNESS 2016;:A2613–A2613. doi:10.1164/ajrccm-conference.2016.193.1_MeetingAbstracts.A2613
28. Hodgson C. A pilot randomised controlled trial of early goal directed mobilisation. Anaesth Intensive Care 2016;44:308.
29. McWilliams D, Jones C, Reeves E, et al. Does enhanced physiotherapy and early mobilisation reduce the degree of muscle loss for patients admitted to critical care? Intensive Care Medicine Experimental 2016;4. doi:10.1186/s40635-016-0100-7
30. Schaller S.J., Waak K., Edrich T., et al. Goal directed early mobilization reduces ICU length of stay and improves functional mobility: An international multi center, randomized, controlled trial (SOMS Trial). Anesth Analg 2016;122:S418. doi:10.1213/01.ane.0000499505.96779.a0
31. Wollersheim T, Malleike J, Haas K, et al. Randomized controlled trial using daily protocol based physiotherapy or protocol based physiotherapy with additional electrical muscle stimulation (EMS) in critically ill patients to prevent intensive care unit (ICU) acquired weakness (ICUAW). Intensive care medicine experimental Conference: 29th annual congress of the european society of intensive care medicine, ESICM 2016 Italy 2016;4. doi:10.1186/s40635-016-0099-9
32. Wright S, Thomas K, Baker C, et al. The extra physiotherapy in critical care (EPICC) multi-centre randomised controlled trial. Intensive care medicine experimental Conference: 29th annual congress of the european society of intensive care medicine, ESICM 2016 Italy 2016;4. doi:10.1186/s40635-016-0099-9
33. Abruzzi F, Azevedo Peixoto Primo J, Marques Filho P, et al. Ultra early mobilization reduces the time of mechanical ventilation and ICU stay. Critical care Conference: 37th international symposium on intensive care and emergency medicine Belgium 2017;21. doi:10.1186/s13054-017-1630-4
34. Bryce H, Hudson A, Law T, et al. Improved physiotherapy outcome measures by the use of cycle ergometry in critical care patients. Critical Care (London, England) 2017;Conference: 37th International Symposium on Intensive Care and Emergency Medicine. Belgium. 21. doi:10.1186/s13054-017-1630-4
35. Carbon N, Wollersheim T, Krebs M, et al. Effects of protocol based physiotherapy and added physiotherapeutic measures on insulin sensitivity in critically ill patients with multiple organ failure. Intensive care medicine experimental Conference: 30th annual congress of the european society of intensive care medicine, ESICM 2017 Austria 2017;5. doi:10.1186/s40635-017-0151-4
36. Fossat G, Baudin F, Coulanges C, et al. Electrical muscle stimulation and bicycling combined to early standard rehabilitation versus early standard rehabilitation alone: impact on global muscle strength at ICU discharge-an open-label, single-centre, assessor-blinded randomised trial. Annals of intensive care Conference: french intensive care society, international congress - reanimation 2017 France 2017;7:9. doi:10.1186/s13613-016-0223-8
37. Gandotra S, Lovato J, Case D, et al. Recovery Trajectories of Critically Ill Patients in a Randomized Controlled Trial of Early Rehabilitation. D15 CRITICAL CARE: DO WE HAVE A CRYSTAL BALL? PREDICTING CLINICAL DETERIORATION AND OUTCOME IN CRITICALLY ILL PATIENTS 2017;:A7019–A7019. doi:10.1164/ajrccm-conference.2017.195.1_MeetingAbstracts.A7019
38. Hickmann J, Castanares-Zapatero D, Deldicque L, et al. Physical therapy during the early course of sepsis is safe and preserves skeletal muscle mass. Annals of intensive care Conference: french intensive care society, international congress - reanimation 2017 France Conference start: 20170111 Conference end: 20170113 2017;7. doi:10.1186/s13613-016-0224-7
39. Kho ME, Molloy AJ, Clarke F, et al. CYCLE Pilot RCT: A Multicenter Feasibility Study of Early in-Bed Cycling Versus Routine Physiotherapy in Medical-Surgical Ventilated Patients. A104 CRITICAL CARE: IMPROVING ICU EXERCISE, REHABILITATION, RECOVERY, AND SURVIVORSHIP 2017;:A2746–A2746. doi:10.1164/ajrccm-conference.2017.195.1_MeetingAbstracts.A2746
40. McWilliams D, Jones C, Atkins G, et al. A Comparison of Early and Enhanced Rehabilitation of Mechanically Ventilated Patients in Critical Care Compared to Standard Care (REHAB): A Single Site Feasibility Randomized Controlled Trial. A104 CRITICAL CARE: IMPROVING ICU EXERCISE, REHABILITATION, RECOVERY, AND SURVIVORSHIP 2017;:A2751–A2751. doi:10.1164/ajrccm-conference.2017.195.1_MeetingAbstracts.A2751
41. Sarfati C, Moore A, Mendialdua P, et al. Study of efficacy on ICU acquired weakness of early standing with the assistance of a tilt table in critically ill patients. Annals of intensive care Conference: french intensive care society, international congress - reanimation 2017 France Conference start: 20170111 Conference end: 20170113 2017;7:206‐207. doi:10.1186/s13613-016-0224-7
42. Wappel SR, Ali O, Serra M, et al. The Effect of an Exercise, Nutrition and Neuromuscular Electrical Stimulation Intervention on Acute Muscle Wasting in Critically Ill Patients Receiving Mechanical Ventilation. A104 CRITICAL CARE: IMPROVING ICU EXERCISE, REHABILITATION, RECOVERY, AND SURVIVORSHIP 2017;:A2747–A2747. doi:10.1164/ajrccm-conference.2017.195.1_MeetingAbstracts.A2747
43. Kho M, Molloy A j., Clarke F j., et al. Outcomes from a Multicentre Pilot Randomized Clinical Trial of Early In-Bed Cycling with Mechanically Ventilated Patients: CYCLE Pilot RCT. C104 CRITICAL CARE: BODY AND MIND IN AND OUT OF THE ICU - SEDATION, DELIRIUM, MOBILIZATION, AND LONG TERM FUNCTIONAL AND COGNITIVE OUTCOMES 2018;:A6031–A6031. doi:10.1164/ajrccm-conference.2018.197.1_MeetingAbstracts.A6031

*Clinical trial registry entry (n=58)*

1. A Study Promoting Critical Illness Recovery in the Elderly - Pilot (NCT02963558)
2. ACT-ICU Study: Activity and Cognitive Therapy in the Intensive Care Unit (NCT01270269)
3. Assessing The Effects of Exercise, Protein, and Electric Stimulation On Intensive Care Unit Patients Outcomes (NCT02509520)
4. Being Awake, Upright and Moving as the Basis for Early ICU Physiotherapy (NCT02301273)
5. CYCLE: A Randomized Clinical Trial of Early In-bed Cycling for Mechanically Ventilated Patients (NCT03471247)
6. Cycling Exercise in Mechanical Ventilation (NCT03581760)
7. Dose of Early Therapeutic Mobility: Does Type or Frequency Matter? (NCT00999011)
8. E-Vent: Electrical muscle stimulation in mechanical Ventilation (ISRCTN35179428)
9. Early Chair Sitting Exercise in Mechanically Ventilated Critically Ill Patients (NCT02021227)
10. Early Cycloergometric Physiotherapy in Critically Ill Patients With Invasive Mechanical Ventilation (NCT02478411)
11. Early Directed Physical Therapy in the Management of Mechanically Ventilated Patients in a Medical Intensive Care Unit (NCT00322010)
12. Early Exercise Training in Critically Ill Patients (NCT00695383)
13. Early Mobilisation in Intensive Care Unit : Interest of Cyclo-ergometry in Patients With Septic Chock (NCT02872792)
14. Early Mobilization and Intensive Rehabilitation in the Critically Ill (NCT02864745)
15. Early Mobilization in Intensive Therapy (NCT01549808)
16. Early Mobilization in the ICU (NCT01777035)
17. Early Neurocognitive Rehabilitation in Intensive Care (NCT02078206)
18. Early Physical Therapy in Patients With Sepsis (NCT01787045)
19. Early Rehabilitation in Critical Illness Survivors (NCT02754505)
20. Early Rehabilitation of COPD Patients in ICU (NCT00628992)
21. Early Rehabilitation Program is Feasible and Safe in ICU in Liver Transplanted Patients (NCT01960868)
22. Effect Of Acute Inflammatory Mediators On Functional Limitations In Patients With Acute Respiratory Failure (NCT01707303)
23. Effect of early mobilisation on respiratory complications following abdominal surgery (ISRCTN28048472)
24. Effects of early, combined endurance and resistance training on mechanically ventilated, critically ill patients – a randomised controlled trial (DRKS00004347)
25. Effects of Neuromuscular Electrical Stimulation on Exercise Capacity in Respiratory Critically Ill Patients (NCT03083652)
26. Efficacy and Safety of a Multicomponent Physical Therapy Program in Mechanically Ventilated Patient With Sepsis (NCT03406494)
27. Electrical Muscle Stimulation (EMS), a Preventive and Therapeutic Tool for Critical Illness Polyneuromyopathy (CIPNM) (NCT00882830)
28. Electrical Muscle Stimulation and Bicycling Combined to Early Standard Rehabilitation in the ICU (NCT02185989)
29. Electro-Neuro-Muscular Stimulation in ICU (NCT02011282)
30. eStimCycle: Early Rehabilitation in Critical Care (ACTRN12612000528853)
31. Exercise in Critically Ill Patients With Sepsis (NCT01364909)
32. Extra physiotherapy in critical care: intensive versus standard physical rehabilitation therapy in the critically ill (ISRCTN20436833)
33. High Protein Intake and Early Exercise in Adult Intensive Care Patients (NCT03469882)
34. Impact of Early Mobilization on Mechanical Ventilation Duration in Intubated Critically Ill Patients (NCT02520193)
35. Impact of the Erigo Machine on Functional Recovery in ICU Patients (NCT02615990)
36. Mobilising Critically Ill patients: Physiological and Functional Outcomes following Early Rehabilitation in Sepsis (ACTRN12610000808044)
37. Mobilization With Neuromuscular Electrical Stimulation in Critical Care Patients (NCT02298114)
38. Neuromuscular Electrical Stimulation in the Critically Ill (NCT02566941)
39. Nutrition and Exercise in Critical Illness (NCT03021902)
40. Progressive Mobility Program and Technology to Improve the Level of Physical Activity and Functionality of ICU Patients (NCT02889146)
41. Progressive Mobilization With Dose Control and Training Load in in Critically Ill Patients (NCT03596853)
42. Project 4B: Lower Extremity Strength Training in ICU Patients (NCT02467023)
43. Rehabilitation After Intensive Care (NCT01770821)
44. Rehabilitation Following Critical Illness (NCT00976807)
45. Safety and Performance of Muscle Activation for Critical Care Patients (NCT01552616)
46. Standardized Rehabilitation for Intensive Care Unit (ICU) Patients With Acute Respiratory Failure (NCT00976833)
47. Study of Safety and Efficacy on Neuromyopathy of Early Standing With the Assistance of Tilt Table in Critically Patients (NCT02047617)
48. Systematic Team Approach to Guide Early Mobilization in Surgical Intensive Care Unit Patients (NCT01363102)
49. TEAM: A Trial of Early Activity and Mobility in ICU (NCT01927510)
50. The EXERCISE trial: Evaluation of exercise rehabilitation for survivors of intensive care (ACTRN12605000776606)
51. The Impact of Early Mobilization Protocol in Patients in the ICU (NCT01769846)
52. Transcutaneous Electric Muscle Stimulation (TEMS) in Septic Patients (NCT01071343)
53. Transcutaneous Electrical Nerve Stimulation Post-thoracic Surgery in a Intensive Care Unit (NCT02438241)
54. Treatment of Critical Illness Polyneuromyopathy (NCT01058421)
55. Treatment of Invasively Ventilated Adults With Early Activity and Mobilisation (NCT03133377)
56. Treatment of Muscle Weakness in Critically Ill Patients (NCT02247895)
57. Use of de game therapy to assess functionality and upper limb muscle strength in critical patients (RBR-6sz5dj)
58. Use of Neuromuscular Electrostimulation (NMES) for Treatment or Prevention of ICU-Associated Weakness (NCT00709124)

*Study Protocol (n=15)*

1. Kayambu G, Boots RJ, Paratz JD. Early rehabilitation in sepsis: a prospective randomised controlled trial investigating functional and physiological outcomes The i-PERFORM Trial (Protocol Article). BMC Anesthesiology 2011;11:21. doi:10.1186/1471-2253-11-21
2. Brummel NE, Jackson JC, Girard TD, et al. A Combined Early Cognitive and Physical Rehabilitation Program for People Who Are Critically Ill: The Activity and Cognitive Therapy in the Intensive Care Unit (ACT-ICU) Trial. Phys Ther 2012;92:1580–92. doi:10.2522/ptj.20110414
3. dos Santos LJ, de Aguiar Lemos F, Bianchi T, et al. Early rehabilitation using a passive cycle ergometer on muscle morphology in mechanically ventilated critically ill patients in the Intensive Care Unit (MoVe-ICU study): study protocol for a randomized controlled trial. Trials 2015;16. doi:10.1186/s13063-015-0914-8
4. Thomas K, Wright SE, Watson G, et al. Extra Physiotherapy in Critical Care (EPICC) Trial Protocol: a randomised controlled trial of intensive versus standard physical rehabilitation therapy in the critically ill. BMJ Open 2015;5:e008035. doi:10.1136/bmjopen-2015-008035
5. Eggmann S, Verra ML, Luder G, et al. Effects of early, combined endurance and resistance training in mechanically ventilated, critically ill patients: a study protocol for a randomised controlled trial. Trials 2016;17:403. doi:10.1186/s13063-016-1533-8
6. Kho ME, Molloy AJ, Clarke F, et al. CYCLE pilot: a protocol for a pilot randomised study of early cycle ergometry versus routine physiotherapy in mechanically ventilated patients. BMJ Open 2016;6:e011659. doi:10.1136/bmjopen-2016-011659
7. Mehrholz J, Thomas S, Burridge JH, et al. Fitness and mobility training in patients with Intensive Care Unit-acquired muscle weakness (FITonICU): study protocol for a randomised controlled trial. Trials 2016;17:559. doi:10.1186/s13063-016-1687-4
8. Nickels MR, Aitken LM, Walsham J, et al. Critical Care Cycling Study (CYCLIST) trial protocol: a randomised controlled trial of usual care plus additional in-bed cycling sessions versus usual care in the critically ill. BMJ Open 2017;7:e017393. doi:10.1136/bmjopen-2017-017393
9. Snelson C, Jones C, Atkins G, et al. A comparison of earlier and enhanced rehabilitation of mechanically ventilated patients in critical care compared to standard care (REHAB): study protocol for a single-site randomised controlled feasibility trial. Pilot and Feasibility Studies 2017;3:19. doi:10.1186/s40814-017-0131-1
10. Thomas S, Mehrholz J. Fitness- und Mobilitätstraining bei Patienten mit auf Intensivstation erworbenem Schwächesyndrom (FITonICU): Protokoll für eine randomisierte kontrollierte Studie. Zeitschrift fur Physiotherapeuten 2017;69:78–84.
11. Wassenaar A, Rood P, Schoonhoven L, et al. The impact of nUrsiNg DEliRium Preventive INnterventions in the Intensive Care Unit (UNDERPIN-ICU): A study protocol for a multi-centre, stepped wedge randomized controlled trial. International Journal of Nursing Studies 2017;68:1–8. doi:10.1016/j.ijnurstu.2016.11.018
12. Lago AF, de Oliveira AS, de Souza HCD, et al. The effects of physical therapy with neuromuscular electrical stimulation in patients with septic shock. Medicine (Baltimore) 2018;97. doi:10.1097/MD.0000000000009736
13. Nielsen AH, Angel S, Egerod I, et al. The effect of diaries written by relatives for intensive care patients on posttraumatic stress (DRIP study): protocol for a randomized controlled trial and mixed methods study. BMC Nursing 2018;17:37. doi:10.1186/s12912-018-0306-y
14. Nydahl P, Diers A, Günther U, et al. PROtokollbasierte MObilisierung auf IntensivstaTIONen [PROtocol-based MObilizaTION on intensive care units : Design of a cluster randomized pilot study]. Med Klin Intensivmed Notfmed 2018;113:581–92. doi:10.1007/s00063-017-0358-x
15. Schujmann DS, Lunardi AC, Fu C. Progressive mobility program and technology to increase the level of physical activity and its benefits in respiratory, muscular system, and functionality of ICU patients: study protocol for a randomized controlled trial. Trials 2018;19. doi:10.1186/s13063-018-2641-4

*Systematic Review/Meta-Analysis (n=4)*

1. Castro-Avila AC, Serón P, Fan E, et al. Effect of Early Rehabilitation during Intensive Care Unit Stay on Functional Status: Systematic Review and Meta-Analysis. PLOS ONE 2015;10:e0130722. doi:10.1371/journal.pone.0130722
2. Connolly B, Salisbury L, O’Neill B, et al. Exercise rehabilitation following intensive care unit discharge for recovery from critical illness. Cochrane Database of Systematic Reviews Published Online First: 2015. doi:10.1002/14651858.CD008632.pub2
3. Elkins M, Dentice R. Inspiratory muscle training facilitates weaning from mechanical ventilation among patients in the intensive care unit: a systematic review. J Physiother 2015;61:125–34. doi:10.1016/j.jphys.2015.05.016
4. Mehrholz J, Pohl M, Kugler J, et al. Physical rehabilitation for critical illness myopathy and neuropathy. Cochrane Database of Systematic Reviews Published Online First: 2015. doi:10.1002/14651858.CD010942.pub2

*Other publication type (n=8)*

1. Melchers P., Maluck A., Suhr L., et al. An early onset rehabilitation program for children and adolescents after traumatic brain injury (TBI): Methods and first results. Restor Neurol Neurosci 1999;14:153–60.
2. Jakob SM, Takala J. Physical and occupational therapy during sedation stops. The Lancet 2009;373:1824–6. doi:10.1016/S0140-6736(09)60866-7
3. Needham DM, Chandolu S, Zanni J. Interruption of sedation for early rehabilitation improves outcomes in ventilated, critically ill adults. Australian Journal of Physiotherapy 2009;55:210. doi:10.1016/S0004-9514(09)70086-8
4. Appleton R. Early Physical and Occupational Therapy in Mechanically Ventilated Medical Patients Improves Return to Independent Functional Status at Hospital Discharge. Journal of the Intensive Care Society 2010;11:202–3. doi:10.1177/175114371001100315
5. Brahmbhatt N, Murugan R, Milbrandt EB. Early mobilization improves functional outcomes in critically ill patients. Critical Care 2010;14:321. doi:10.1186/cc9262
6. Charet GP. InBox: PATIENT CARE. To Reduce ICU Stays, Get Patients Moving. H&HN: Hospitals & Health Networks 2010;84:14–14.
7. Felten-Barentsz KM, Haans AJC, Slutsky AS, et al. Feasibility and Safety of Hydrotherapy in Critically Ill Ventilated Patients. Am J Respir Crit Care Med 2015;191:476–7. doi:10.1164/rccm.201408-1559LE
8. Unknown Author. Early, goal-directed mobilisation in the surgical intensive care unit: a randomised controlled trial. New Zealand Medical Journal 2016;129:102–102.

*No RCT (n=10)*

1. de Morton NA, Keating JL, Berlowitz DJ, et al. Additional exercise does not change hospital or patient outcomes in older medical patients: a controlled clinical trial. Australian Journal of Physiotherapy 2007;53:105–11. doi:10.1016/S0004-9514(07)70043-0
2. Morris PE, Goad A, Thompson C, et al. Early intensive care unit mobility therapy in the treatment of acute respiratory failure*. Critical Care Medicine 2008;36:2238. doi:10.1097/CCM.0b013e318180b90e
3. Needham DM, Korupolu R, Zanni JM, et al. Early Physical Medicine and Rehabilitation for Patients With Acute Respiratory Failure: A Quality Improvement Project. Archives of Physical Medicine and Rehabilitation 2010;91:536–42. doi:10.1016/j.apmr.2010.01.002
4. Caruso FCR, Arena R, Mendes RG, et al. Heart rate autonomic responses during deep breathing and walking in hospitalised patients with chronic heart failure. Disability and Rehabilitation 2011;33:751–7. doi:10.3109/09638288.2010.511420
5. Hanekom SD, Louw Q, Coetzee A. The way in which a physiotherapy service is structured can improve patient outcome from a surgical intensive care: a controlled clinical trial. Critical Care 2012;16:R230. doi:10.1186/cc11894
6. Paratz JD, Stockton K, Plaza A, et al. Intensive exercise after thermal injury improves physical, functional, and psychological outcomes: Journal of Trauma and Acute Care Surgery 2012;73:186–94. doi:10.1097/TA.0b013e31824baa52
7. Parry SM, Berney S, Warrillow S, et al. Functional electrical stimulation with cycling in the critically ill: A pilot case-matched control study. Journal of Critical Care 2014;29:695.e1-695.e7. doi:10.1016/j.jcrc.2014.03.017
8. Wang YT, Haines TP, Ritchie P, et al. Early mobilization on continuous renal replacement therapy is safe and may improve filter life. Critical Care 2014;18:R161. doi:10.1186/cc14001
9. Floyd S, Craig SW, Topley D, et al. Evaluation of a Progressive Mobility Protocol in Postoperative Cardiothoracic Surgical Patients. Dimensions of Critical Care Nursing 2016;35:277. doi:10.1097/DCC.0000000000000197
10. Turon M, Fernandez-Gonzalo S, Jodar M, et al. Feasibility and safety of virtual-reality-based early neurocognitive stimulation in critically ill patients. Ann Intensive Care 2017;7. doi:10.1186/s13613-017-0303-4

*Ineligible language (n=2)*

1. Hui K, Haiyan H. Effect observation on four stage early activity and rehabilitation exercise therapy for prevention of patients with ICU acquired weakness. Chinese Nursing Research 2016;30:2202–5. doi:10.3969/j.issn.1009-6493.2016.18.009
2. Zhu C, Liu B, Yang T, et al. [Effect of early rehabilitation physiotherapy on muscle quality and function in critically ill patients]. Zhonghua Wei Zhong Bing Ji Jiu Yi Xue 2018;30:569–72. doi:10.3760/cma.j.issn.2095-4352.2018.06.013

*Population not eligible (n=47)*

1. Hayes MJ, Morris GK, Hampton JR. Comparison of Mobilization after Two and Nine Days in Uncomplicated Myocardial Infarction. Br Med J 1974;3:10–3.
2. Stiller K, Montarello J, Wallace M, et al. Efficacy of Breathing and Coughing Exercises in the Prevention of Pulmonary Complications After Coronary Artery Surgery. Chest 1994;105:741–7. doi:10.1378/chest.105.3.741
3. Olsén MF, Hahn I, Nordgren S, et al. Randomized controlled trial of prophylactic chest physiotherapy in major abdominal surgery. BJS 1997;84:1535–8. doi:10.1111/j.1365-2168.1997.02828.x
4. Weiner P, Zeidan F, Zamir D, et al. Prophylactic Inspiratory Muscle Training in Patients Undergoing Coronary Artery Bypass Graft. World Journal of Surgery 1998;22:427–31. doi:10.1007/s002689900410
5. Arthur HM, Daniels C, McKelvie R, et al. Effect of a preoperative intervention on preoperative and postoperative outcomes in low-risk patients awaiting elective coronary artery bypass graft surgery. A randomized, controlled trial. Ann Intern Med 2000;133:253–62.
6. Patman S, Sanderson D, Blackmore M. Physiotherapy following cardiac surgery: Is it necessary during the intubation period? Australian Journal of Physiotherapy 2001;47:7–16. doi:10.1016/S0004-9514(14)60294-4
7. Delaney CP, Zutshi M, Senagore AJ, et al. Prospective, Randomized, Controlled Trial Between a Pathway of Controlled Rehabilitation With Early Ambulation and Diet and Traditional Postoperative Care After Laparotomy and Intestinal Resection: Diseases of the Colon & Rectum 2003;46:851–9. doi:10.1007/s10350-004-6672-4
8. Mackay MR, Ellis E, Johnston C. Randomised clinical trial of physiotherapy after open abdominal surgery in high risk patients. Australian Journal of Physiotherapy 2005;51:151–9. doi:10.1016/S0004-9514(05)70021-0
9. Chiang L-L, Wang L-Y, Wu C-P, et al. Effects of Physical Training on Functional Status in Patients With Prolonged Mechanical Ventilation. Phys Ther 2006;86:1271–81. doi:10.2522/ptj.20050036
10. Templeton M, Palazzo MGA. Chest physiotherapy prolongs duration of ventilation in the critically ill ventilated for more than 48 hours. Intensive Care Med 2007;33:1938–45. doi:10.1007/s00134-007-0762-4
11. Herdy AH, Marcchi PLB, Vila A, et al. Pre- and Postoperative Cardiopulmonary Rehabilitation in Hospitalized Patients Undergoing Coronary Artery Bypass Surgery: A Randomized Controlled Trial. American Journal of Physical Medicine & Rehabilitation 2008;87:714. doi:10.1097/PHM.0b013e3181839152
12. Albert NM, Gillinov AM, Lytle BW, et al. A randomized trial of massage therapy after heart surgery. Heart & Lung 2009;38:480–90. doi:10.1016/j.hrtlng.2009.03.001
13. Burtin C, Clerckx B, Robbeets C, et al. Early exercise in critically ill patients enhances short-term functional recovery*. Critical Care Medicine 2009;37:2499–505. doi:10.1097/CCM.0b013e3181a38937
14. Forgiarini Junior LA, Carvalho AT de, Ferreira T de S, et al. Physical therapy in the immediate postoperative period after abdominal surgery. Jornal Brasileiro de Pneumologia 2009;35:445–59. doi:10.1590/S1806-37132009000500011
15. Jarden M, Baadsgaard MT, Hovgaard DJ, et al. A randomized trial on the effect of a multimodal intervention on physical capacity, functional performance and quality of life in adult patients undergoing allogeneic SCT. Bone Marrow Transplantation 2009;43:725–37. doi:10.1038/bmt.2009.27
16. Patman S, Jenkins S, Stiller K. Physiotherapy does not prevent, or hasten recovery from, ventilator-associated pneumonia in patients with acquired brain injury. Intensive Care Med 2009;35:258–65. doi:10.1007/s00134-008-1278-2
17. Stein R, Maia CP, Silveira AD, et al. Inspiratory Muscle Strength as a Determinant of Functional Capacity Early After Coronary Artery Bypass Graft Surgery. Archives of Physical Medicine and Rehabilitation 2009;90:1685–91. doi:10.1016/j.apmr.2009.05.010
18. Mendes RG, Simões RP, Costa FDSM, et al. Short-term supervised inpatient physiotherapy exercise protocol improves cardiac autonomic function after coronary artery bypass graft surgery – a randomised controlled trial. Disability and Rehabilitation 2010;32:1320–7. doi:10.3109/09638280903483893
19. Pattanshetty RB, Gaude GS. Effect of multimodality chest physiotherapy in prevention of ventilator-associated pneumonia: A randomized clinical trial. Indian J Crit Care Med 2010;14:70–6. doi:10.4103/0972-5229.68218
20. Reeve JC, Nicol K, Stiller K, et al. Does physiotherapy reduce the incidence of postoperative pulmonary complications following pulmonary resection via open thoracotomy? A preliminary randomised single-blind clinical trial. Eur J Cardiothorac Surg 2010;37:1158–66. doi:10.1016/j.ejcts.2009.12.011
21. Routsi C, Gerovasili V, Vasileiadis I, et al. Electrical muscle stimulation prevents critical illness polyneuromyopathy: a randomized parallel intervention trial. Critical Care 2010;14:R74. doi:10.1186/cc8987
22. Troosters T, Probst VS, Crul T, et al. Resistance Training Prevents Deterioration in Quadriceps Muscle Function During Acute Exacerbations of Chronic Obstructive Pulmonary Disease. Am J Respir Crit Care Med 2010;181:1072–7. doi:10.1164/rccm.200908-1203OC
23. Chen S, Su C-L, Wu Y-T, et al. Physical training is beneficial to functional status and survival in patients with prolonged mechanical ventilation. Journal of the Formosan Medical Association 2011;110:572–9. doi:10.1016/j.jfma.2011.07.008
24. Pattanshetty RB, Gaude GS. Effect of multimodality chest physiotherapy on the rate of recovery and prevention of complications in patients with mechanical ventilation: a prospective study in medical and surgical intensive care units. Indian J Med Sci 2011;65:175–85. doi:10.4103/0019-5359.106608
25. Chen Y-H, Lin H-L, Hsiao H-F, et al. Effects of Exercise Training on Pulmonary Mechanics and Functional Status in Patients With Prolonged Mechanical Ventilation. Respiratory Care 2012;57:727–34. doi:10.4187/respcare.01341
26. Hirschhorn AD, Richards DAB, Mungovan SF, et al. Does the mode of exercise influence recovery of functional capacity in the early postoperative period after coronary artery bypass graft surgery? A randomized controlled trial. Interact CardioVasc Thorac Surg 2012;15:995–1003. doi:10.1093/icvts/ivs403
27. Jackson JC, Ely EW, Morey MC, et al. Cognitive and physical rehabilitation of intensive care unit survivors: Results of the RETURN randomized controlled pilot investigation*. Critical Care Medicine 2012;40:1088. doi:10.1097/CCM.0b013e3182373115
28. Karatzanos E, Gerovasili V, Zervakis D, et al. Electrical Muscle Stimulation: An Effective Form of Exercise and Early Mobilization to Preserve Muscle Strength in Critically Ill Patients. Critical Care Research and Practice 2012;2012:1–8. doi:10.1155/2012/432752
29. Yohannan SK, Tufaro PA, Hunter H, et al. The Utilization of Nintendo® WiiTM During Burn Rehabilitation: A Pilot Study. J Burn Care Res 2012;33:36–45. doi:10.1097/BCR.0b013e318234d8ef
30. Lee S-M, Kang S-B, Jang J-H, et al. Early rehabilitation versus conventional care after laparoscopic rectal surgery: a prospective, randomized, controlled trial. Surg Endosc 2013;27:3902–9. doi:10.1007/s00464-013-3006-4
31. Connolly B, Thompson A, Douiri A, et al. Exercise-based rehabilitation after hospital discharge for survivors of critical illness with intensive care unit–acquired weakness: A pilot feasibility trial. Journal of Critical Care 2015;30:589–98. doi:10.1016/j.jcrc.2015.02.002
32. Jones C, Eddleston J, McCairn A, et al. Improving rehabilitation after critical illness through outpatient physiotherapy classes and essential amino acid supplement: A randomized controlled trial. Journal of Critical Care 2015;30:901–7. doi:10.1016/j.jcrc.2015.05.002
33. Peixoto TCA, Begot I, Bolzan DW, et al. Early Exercise-Based Rehabilitation Improves Health-Related Quality of Life and Functional Capacity After Acute Myocardial Infarction: A Randomized Controlled Trial. Canadian Journal of Cardiology 2015;31:308–13. doi:10.1016/j.cjca.2014.11.014
34. Trevisan MD, Lopes DGC, de Mello RGB, et al. Alternative Physical Therapy Protocol Using a Cycle Ergometer During Hospital Rehabilitation of Coronary Artery Bypass Grafting: a Clinical Trial. Braz J Cardiovasc Surg 2015;30:615–9. doi:10.5935/1678-9741.20150085
35. Walsh TS, Salisbury LG, Merriweather JL, et al. Increased Hospital-Based Physical Rehabilitation and Information Provision After Intensive Care Unit Discharge: The RECOVER Randomized Clinical Trial. JAMA Intern Med 2015;175:901–10. doi:10.1001/jamainternmed.2015.0822
36. Yosef‐Brauner O, Adi N, Shahar TB, et al. Effect of physical therapy on muscle strength, respiratory muscles and functional parameters in patients with intensive care unit-acquired weakness. The Clinical Respiratory Journal 2015;9:1–6. doi:10.1111/crj.12091
37. Karadas C, Ozdemir L. The effect of range of motion exercises on delirium prevention among patients aged 65 and over in intensive care units. Geriatric Nursing 2016;37:180–5. doi:10.1016/j.gerinurse.2015.12.003
38. Machado A dos S, Pires-Neto RC, Carvalho MTX, et al. Effects that passive cycling exercise have on muscle strength, duration of mechanical ventilation, and length of hospital stay in critically ill patients: a randomized clinical trial. Jornal Brasileiro de Pneumologia 2017;43:134–9. doi:10.1590/s1806-37562016000000170
39. Maffei P, Wiramus S, Bensoussan L, et al. Intensive Early Rehabilitation in the Intensive Care Unit for Liver Transplant Recipients: A Randomized Controlled Trial. Archives of Physical Medicine and Rehabilitation 2017;98:1518–25. doi:10.1016/j.apmr.2017.01.028
40. Tariq MI, Khan AA, Khalid Z, et al. Effect of Early ≤ 3 Mets (Metabolic Equivalent of Tasks) of Physical Activity on Patient’s Outcome after Cardiac Surgery. 2017;27:6.
41. Zhao J, Yao L, Wang C, et al. The effects of cognitive intervention on cognitive impairments after intensive care unit admission. Neuropsychological Rehabilitation 2017;27:301–17. doi:10.1080/09602011.2015.1078246
42. dos Santos FV, Jr GC, Vieira L, et al. Neuromuscular electrical stimulation combined with exercise decreases duration of mechanical ventilation in ICU patients: A randomized controlled trial. Physiotherapy Theory and Practice 2018;0:1–9. doi:10.1080/09593985.2018.1490363
43. Fontes Cerqueira TC, de Cerqueira Neto ML, Cacau L de AP, et al. Ambulation capacity and functional outcome in patients undergoing neuromuscular electrical stimulation after cardiac valve surgery. Medicine (Baltimore) 2018;97. doi:10.1097/MD.0000000000013012
44. Jahangirifard A, Razavi M, Ahmadi ZH, et al. Effect of TENS on Postoperative Pain and Pulmonary Function in Patients Undergoing Coronary Artery Bypass Surgery. Pain Management Nursing 2018;19:408–14. doi:10.1016/j.pmn.2017.10.018
45. McWilliams D, Jones C, Atkins G, et al. Earlier and enhanced rehabilitation of mechanically ventilated patients in critical care: A feasibility randomised controlled trial. Journal of Critical Care 2018;44:407–12. doi:10.1016/j.jcrc.2018.01.001
46. Sarfati C, Moore A, Pilorge C, et al. Efficacy of early passive tilting in minimizing ICU-acquired weakness: A randomized controlled trial. Journal of Critical Care 2018;46:37–43. doi:10.1016/j.jcrc.2018.03.031
47. Verceles AC, Wells CL, Sorkin JD, et al. A multimodal rehabilitation program for patients with ICU acquired weakness improves ventilator weaning and discharge home. Journal of Critical Care 2018;47:204–10. doi:10.1016/j.jcrc.2018.07.006

*Intervention not eligible (n=12)*

1. Nava S. Rehabilitation of patients admitted to a respiratory intensive care unit. Archives of Physical Medicine and Rehabilitation 1998;79:849–54. doi:10.1016/S0003-9993(98)90369-0
2. Zanotti E, Felicetti G, Maini M, et al. Peripheral Muscle Strength Training in Bed-Bound Patients With COPD Receiving Mechanical Ventilation: Effect of Electrical Stimulation. Chest 2003;124:292–6. doi:10.1378/chest.124.1.292
3. Susa A, Roveran A, Bocchi A, et al. [FastTrack approach to major colorectal surgery]. Chir Ital 2004;56:817–24.
4. Caruso P, Denari SD, Ruiz SA, et al. Inspiratory muscle training is ineffective in mechanically ventilated critically ill patients. Clinics 2005;60:479–84. doi:10.1590/S1807-59322005000600009
5. Porta R, Vitacca M, Gilè LS, et al. Supported Arm Training in Patients Recently Weaned From Mechanical Ventilation. Chest 2005;128:2511–20. doi:10.1378/chest.128.4.2511
6. Cader SA, de Vale RGS, Castro JC, et al. Inspiratory muscle training improves maximal inspiratory pressure and may assist weaning in older intubated patients: a randomised trial. Journal of Physiotherapy 2010;56:171–7. doi:10.1016/S1836-9553(10)70022-9
7. Jackson JC, Girard TD, Gordon SM, et al. Long-term Cognitive and Psychological Outcomes in the Awakening and Breathing Controlled Trial. Am J Respir Crit Care Med 2010;182:183–91. doi:10.1164/rccm.200903-0442OC
8. Médrinal C, Lebret M, Bousta M, et al. Effets de la station assise au bord du lit du patient intubé et ventilé. Kinésithérapie, la revue 2013;13:43–9.
9. Collings N, Cusack R. A repeated measures, randomised cross-over trial, comparing the acute exercise response between passive and active sitting in critically ill patients. BMC Anesthesiology 2015;15:1. doi:10.1186/1471-2253-15-1
10. Moss M, Nordon-Craft A, Malone D, et al. A Randomized Trial of an Intensive Physical Therapy Program for Patients with Acute Respiratory Failure. Am J Respir Crit Care Med 2016;193:1101–10. doi:10.1164/rccm.201505-1039OC
11. Neumeier A, Nordon-Craft A, Malone D, et al. Prolonged acute care and post-acute care admission and recovery of physical function in survivors of acute respiratory failure: a secondary analysis of a randomized controlled trial. Critical Care 2017;21:190. doi:10.1186/s13054-017-1791-1
12. Wright SE, Thomas K, Watson G, et al. Intensive versus standard physical rehabilitation therapy in the critically ill (EPICC): a multicentre, parallel-group, randomised controlled trial. Thorax 2018;73:213–21. doi:10.1136/thoraxjnl-2016-209858

*Comparator not eligible (n=12)*

1. Patel BK, Pohlman AS, Hall JB, et al. Impact of Early Mobilization on Glycemic Control and ICU-Acquired Weakness in Critically Ill Patients Who Are Mechanically Ventilated. Chest 2014;146:583–9. doi:10.1378/chest.13-2046
2. Kho ME, Truong AD, Zanni JM, et al. Neuromuscular electrical stimulation in mechanically ventilated patients: A randomized, sham-controlled pilot trial with blinded outcome assessment. J Crit Care 2015;30:32–9. doi:10.1016/j.jcrc.2014.09.014
3. Coutinho WM, Santos LJ dos, Fernandes J, et al. Acute effect of the use of cycle ergometer during physical therapy treatment in mechanically ventilated critically ill patients. Fisioterapia e Pesquisa 2016;23:278–83. doi:10.1590/1809-2950/15549123032016
4. Akar O, Günay E, Ulasli SS, et al. Efficacy of neuromuscular electrical stimulation in patients with COPD followed in intensive care unit. The Clinical Respiratory Journal 2017;11:743–50. doi:10.1111/crj.12411
5. dall’Acqua A, Sachetti A, Santos L, et al. Use of neuromuscular electrical stimulation to preserve the thickness of abdominal and chest muscles of critically ill patients: A randomized clinical trial. Journal of Rehabilitation Medicine 2017;49:40–8. doi:10.2340/16501977-2168
6. Shen S-Y, Lee C-H, Lin R-L, et al. Electric Muscle Stimulation for Weaning from Mechanical Ventilation in Elder Patients with Severe Sepsis and Acute Respiratory Failure – A Pilot Study. International Journal of Gerontology 2017;11:41–5. doi:10.1016/j.ijge.2017.01.001
7. Fossat G, Baudin F, Courtes L, et al. Effect of In-Bed Leg Cycling and Electrical Stimulation of the Quadriceps on Global Muscle Strength in Critically Ill Adults: A Randomized Clinical Trial. JAMA 2018;320:368–78. doi:10.1001/jama.2018.9592
8. Gandotra S, Lovato J, Case D, et al. Physical Function Trajectories in Survivors of Acute Respiratory Failure. Annals ATS Published Online First: 20 December 2018. doi:10.1513/AnnalsATS.201806-375OC
9. Hickmann CE, Castanares-Zapatero D, Deldicque L, et al. Impact of Very Early Physical Therapy During Septic Shock on Skeletal Muscle: A Randomized Controlled Trial. Crit Care Med 2018;46:1436–43. doi:10.1097/CCM.0000000000003263
10. Médrinal C, Combret Y, Prieur G, et al. Comparison of exercise intensity during four early rehabilitation techniques in sedated and ventilated patients in ICU: a randomised cross-over trial. Crit Care 2018;22. doi:10.1186/s13054-018-2030-0
11. Winkelman C, Sattar A, Momotaz H, et al. Dose of Early Therapeutic Mobility: Does Frequency or Intensity Matter? Biological Research For Nursing 2018;20:522–30. doi:10.1177/1099800418780492
12. Wolfe KS, Patel BK, MacKenzie EL, et al. Impact of Vasoactive Medications on ICU-Acquired Weakness in Mechanically Ventilated Patients. Chest 2018;154:781–7. doi:10.1016/j.chest.2018.07.016

*Outcome reporting not eligible (n=1)*

1. Suwardianto H, Prasetyo A, Utami R. Effects of physical-cognitive therapy (PCT) on criticaly ill patients in intensive care unit. Hiroshima Journal of Medical Sciences 2018;67:63–9.
