# Supplementary material for: Systematic early versus late mobilization or standard early mobilization in mechanically ventilated adult ICU patients: systematic review and meta-analysis
Source: Crit Care. 2021 Jan 6;25:16. doi: 10.1186/s13054-020-03446-9 (PMC7789482; doi:10.1186/s13054-020-03446-9)
Supplement: Supplementary file 2 — Additional file 2. Risk of Bias assessment details. [file 13054_2020_3446_MOESM2_ESM.docx]

**Additional File 2**

**Risk of Bias Assessment**


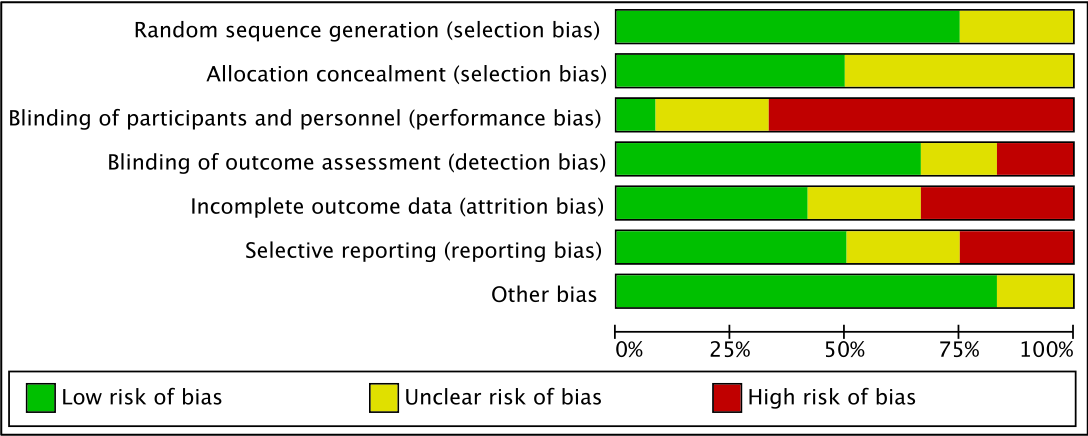
Summary

Assessment details

| **Study** | **Sequence Generation** | **Allocation Concealment** | **Blinding of Patients and Personnel** | **Blinding of Outcome Assessors** | **Incomplete Outcome Data** | **Selective Reporting** | **Other Risk of Bias** | **Overall Assessment*** |
| --- | --- | --- | --- | --- | --- | --- | --- | --- |
| Schweickert et al. 2009 | Low | Low | High | Low | Low | Low | - | good |
| Comment |  |  | "the nature of the intervention prevented any blinding from patient and health-care providers" |  |  |  |  |  |
| Dantas et al. 2012 | Unclear | Unclear | Unclear | Unclear | Low | Unclear | Present | poor |
| Comment | Randomization strategy not stated. | Allocation concealment strategy not stated. | No information on blinding of participants available. | No information on blinding of outcome assessors available. |  | No protocol available. | Extreme baseline imbalance (age and gender). |  |
| Denehy et al. 2013 | Low | Low | High | Low | Unclear | High | - | poor |
| Comment |  |  | "maintained (single) blinding" |  | Different n for each outcome measure reported (Table 3). | No information provided for some prespecified outcomes and time points. |  |  |
| Brummel et al. 2014 | Low | Low | High | Low | High | High | - | poor |
| Comment |  |  | "inability to blind patients or those performing the interventions" |  | Follow-up occurred less frequently in the cognitive plus physical therapy group because of withdrawals. | No information provided for some prespecified outcomes and time point. |  |  |
| Dong et al. 2014 | Unclear | Unclear | High | Unclear | Unclear | Unclear | - | poor |
| Comment | Randomization strategy not stated. | Allocation concealment not mentioned. | "this study [...] is not double blinded" | No information on blinding of outcome assessors available. | No information on attrition available. | No protocol available. |  |  |
| Kayambu et al. 2015 | Low | Unclear | Low | Low | High | High | - | poor |
| Comment |  | Allocation concealment strategy not stated. |  |  | Differentially more losses in intervention group. | No information provided for some prespecified outcomes and time points. |  |  |
| Dong et al. 2016 | Low | Unclear | High | High | Low | Unclear | - | poor |
| Comment |  | Allocation concealment not mentioned. | "Blinding could not be performed in this randomized study" | "Blinding could not be performed in this randomized study" |  | Outcomes not clearly prespecified. No muscle strength or functional mobility outcome reported. |  |  |
| Fischer et al. 2016 | Low | Unclear | Unclear | High | Unclear | Low | - | poor |
| Comment |  | Allocation concealment not mentioned. | Blinding attempted but potentially broken, depends on communication with patients. | "Nonblinded assessors performed the ultrasound scans, [...]"; Results are at high risk of bias due to unblinded performance of ultrasound scans. | Significant attrition (>30%), but no missing outcome data for outcomes of interest. Intention-to-treat analysis, no imputation used. |  |  |  |
| Hodgson et al. 2016 | Unclear | Low | High | Low | Low | Low | Present | poor |
| Comment | Randomization not mentioned. |  | "all clinicians involved in their care were aware of study-group assignments"; Specifically mentioned as assessor-blinded, no mentioning of blinding of patients. |  |  |  | Potential issues related to study design: imbalance in group size (21 vs. 29 under randomized allocation). |  |
| Morris et al. 2016 | Low | Unclear | High | Low | High | Low | - | poor |
| Comment |  | Allocation concealment not mentioned. | Specifically mentioned as assessor-blinded, no mentioning of blinding of patients. |  | High amount of missing data at several timepoints. |  |  |  |
| Schaller et al. 2016 | Low | Low | Unclear | Low | High | Low | - | fair |
| Comment |  |  | "Patients were not made aware of their assignment." |  | Significant loss to follow-up (38% of patients). Multiple imputation for SF-36 at 3 months performed as sensitivity analysis. |  |  |  |
| Eggmann et al. 2018 | Low | Low | High | Low | Low | Low | - | good |
| Comment |  |  | "Blinding the responsible ICU staff was impossible";  "blinding of participants and physiotherapists was impossible." |  |  |  |  |  |

**blinding of personnel not considered for the overall assessment, as judged almost impossible to perform.*
