# Supplementary material for: Systematic early versus late mobilization or standard early mobilization in mechanically ventilated adult ICU patients: systematic review and meta-analysis
Source: Crit Care. 2021 Jan 6;25:16. doi: 10.1186/s13054-020-03446-9 (PMC7789482; doi:10.1186/s13054-020-03446-9)
Supplement: Supplementary file 3 — Additional file 3. Full results. [file 13054_2020_3446_MOESM3_ESM.docx]

**Additional File 3**

**Detailed Results**

Muscle strength

| **Outcome** | **Comparison** | **Study** | **Time point** | **Comparator (Mean (SD)/Median (IQR))** | **Intervention [Mean (SD)/Median (IQR))** | **Reported p-Value** |
| --- | --- | --- | --- | --- | --- | --- |
| **MRC Sum Score (MRC-SS)*** | (i) | Schweickert et al. 2009 | Hospital discharge | 48 (0-58) | 52 (25-58) | 0.38 |
|  | (ii) | Dantas et al. 2012 | ICU discharge | 40.3 (10.5) | 55.86 (4.4) | <0.001 |
|  |  | Kayambu et al. 2015 | ICU discharge | 47.3 (13.6) | 51.9 (10.5) | 0.24 |
|  |  | Hodgson et al. 2016 | ICU discharge | 45.2 (13.2) | 50.4 (7.5) | 0.1 |
|  |  | Eggmann et al. 2018 | ICU discharge | 44.4 (11.7) | 42.4 (13.1) | 0.46 |
| **Incidence of ICU-Acquired Weakness (ICUAW)*** | (i) | Schweickert et al. 2009 | Hospital discharge | 27/55 (49.1%) | 15/49 (30.6%) | 0.09 |
|  | (ii) | Denehy et al. 2013 | Hospital discharge | 13/76 (17.1%) | 16/74 (21.6%) | - |
|  |  | Hodgson et al. 2016 | ICU discharge | 10/20 (50%) | 7/25 (28%) | 0.13 |
|  |  | Schaller et al. 2016 | Hospital discharge | 51/96 (53.1%) | 50/104 (48.1%) | 0.95 |
| **Hand-held dynamometry (in lb)** | (i) | Morris et al. 2016** | ICU discharge | 22.8 (10.5) | 20.3 (10.2) | 0.16 |
|  |  |  | Hospital discharge | 23.9 (10.7) | 23.7 (10.7) | 0.90 |
|  |  |  | 2 month follow-up | 28.0 (10.5) | 28.5 (10.5) | 0.76 |
|  |  |  | 4 month follow-up | 29.6 (10.4) | 28.8 (10.6) | 0.63 |
|  |  |  | 6 month follow-up | 30.8 (10.5) | 31.1 (10.4) | 0.82 |
| **Handgrip strength (in kg)** | (i) | Schweickert et al. 2009 | Hospital discharge | 35 (0-57) | 39 (10-58) | 0.67 |
|  |  | Morris et al. 2016** | ICU discharge | 20.9 (10.5) | 20 (10.1) | 0.6 |
|  |  |  | Hospital discharge | 24.3 (10.4) | 22.6 (10.4) | 0.25 |
|  |  |  | 2 month follow-up | 26.0 (9.4) | 27.2 (9.8) | 0.43 |
|  |  |  | 4 month follow-up | 27.2 (10.1) | 29.0 (10.5) | 0.25 |
|  |  |  | 6 month follow-up | 27.2 (11.0) | 29.3 (10.9) | 0.23 |
|  | (ii) | Eggmann et al. 2018 | ICU discharge | 19.6 (13.6) | 20.5 (12.6) | 0.78 |

**=Prioritized by ICU Experts as most clinically relevant priority outcomes; **=Standard deviation calculated from 95% confidence interval; (i)=systematic early vs. late mobilization; (ii)=systematic early vs. standard early mobilization.*

*Figure 1: Fixed- and random-effects meta-analysis of the mean differences [95% confidence interval] in MRC Sum Score (MRC-SS) at ICU discharge between systematic early mobilization and standard early mobilization: Sensitivity analysis excluding the study by Dantas et al. 2012 for high baseline imbalance in MRC-SS.*

Functional mobility

| **Outcome** | **Comparison** | **Study** | **Time point** | **Comparator (Mean (SD)/Median (IQR) or  n (%))** | **Intervention [Mean (SD)/Median (IQR) or  n (%))** | **Reported p-Value** |
| --- | --- | --- | --- | --- | --- | --- |
| **Barthel Index** | (i) | Schweickert et al. 2009 | Hospital discharge | 55 (0-85) | 75 (7.5-95) | 0.05 |
| **Activities of Daily Living (ADL)** | (i) | Schweickert et al. 2009 | ICU discharge | 0 (0-5) | 3 (0-5) | 0.15 |
|  |  |  | Hospital discharge | 4 (0-6) | 6 (0-6) | 0.06 |
|  | (ii) | Brummel et al. 2014** | Hospital discharge | 1 (0-2.8) | 0.5 (0-4.5) / 3 (1-6) | 0.25 |
|  |  |  | 3 month follow-up | 0 (0-0) | 0 (0-1) / 0 (0-2) | 0.69 |
|  |  | Hodgson et al. 2016 | 6 month follow-up | 7 (1.3) | 6.5 (1.9) | 0.81 |
| **Functional Independence Measure (FIM)** | (ii) | Schaller et al. 2016*** | ICU discharge | 3 (1-4) | 4 (2-5) | 0.009 |
|  |  |  | Hospital discharge | 5 (2-8) | 8 (4-8) | 0.0002 |
|  |  | Eggmann et al. 2018 | ICU discharge | 28.5 (19.5-41.5) | 28.5 (21-42) | 0.79 |
|  |  |  | Hospital discharge | 99 (24) | 101 (22) | 0.66 |
| **Physical Function in the ICU Test (PFIT)** | (ii) | Denehy et al. 2013 | ICU discharge | 8 (1.5) | 7.7 (1.7) | - |
|  |  | Kayambu et al. 2015 | ICU discharge | 5.4 (1.7) | 5.6 (2.1) | 0.61 |
|  |  | Hodgson et al. 2016 | ICU discharge | 7.4 (3.6) | 7.4 (3.6) | 0.83 |
| **Timed up-and-go test (TUG; in sec)** | (ii) | Denehy et al. 2013 | ICU discharge | 36.1 (42.9) | 41.1 (43.2) | - |
|  |  |  | Hospital discharge | 12.9 (6.6) | 18.8 (24.5) | - |
|  |  |  | 3 month follow-up | 11.6 (11.2) | 12.2 (10.0) | - |
|  |  |  | 6 month follow-up | 12.9 (17.9) | 9.8 (5.1) | - |
|  |  |  | 12 month follow-up | 14.2 (24.7) | 10.3 (6.2) | - |
|  |  | Brummel et al. 2014** | Hospital discharge | 33 (18.5-68.5) | 16 (12-22) / 17 (11-27) | 0.2 |
|  |  |  | 3 month follow-up | 8 (7.5-13.5) | 10 (8-13) / 11 (9-13) | 0.79 |
|  |  | Eggmann et al. 2018 | Hospital discharge | 16 (10.3-29) | 19.5 (11.5-25) | 0.54 |
| **6-minute walking test (6MWT; in meters)*** | (ii) | Denehy et al. 2013 | ICU discharge | 187.9 (126.1) | 146.4 (79.4) | - |
|  |  |  | Hospital discharge | 266.7 (136.8) | 244.2 (124.0) | - |
|  |  |  | 3 month follow-up | 382.1 (139.4) | 384.5 (147.9) | - |
|  |  |  | 6 month follow-up | 402.4 (166.6) | 394.2 (156.2) | - |
|  |  |  | 12 month follow-up | 409.6 (158.5) | 433.8 (150.7) | - |
|  |  | Eggmann et al. 2018 | Hospital discharge | 246 (167) | 223 (133) | 0.45 |
| **Distance walked without assistance (in meters)** | (i) | Schweickert et al. 2009 | Hospital discharge | 0 (0-30.4) | 33.4 (0-91.4) | 0.004 |
| **Time to first time out of bed (in days)** | (i) | Schweickert et al. 2009 |  | 6.6 (4.2-8.3) | 1.7 (1.1-3) | <0.0001 |
|  | (ii) | Dong et al. 2014 |  | 14.9 (4.7) | 3.8 (1.2) | <0.01 |
|  |  | Eggmann et al. 2018 |  | 5 (2-7) | 4 (2-7) | 0.45 |
| **Time to standing (in days)** | (i) | Schweickert et al. 2009 |  | 6 (4.5-8.9) | 3.2 (1.5-5.6) | <0.0001 |
|  | (ii) | Hodgson et al. 2016 |  | 3 (2.4-4.5) | 3 (2-6) | 0.88 |
|  |  | Eggmann et al. 2018 |  | 7.5 (3-14) | 10 (n=3) | - |
| **Time to walking (in days)*** | (i) | Schweickert et al. 2009 |  | 7.3 (4.9-9.6) | 3.8 (1.9-5.8) | <0.0001 |
|  | (ii) | Hodgson et al. 2016 |  | 6 (3-8) | 6 (3-12) | 0.97 |
|  |  | Eggmann et al. 2018 |  | 23 (n=2) | 8 (n=1) | - |
| **Patients returning to independence from assistance*** | (i) | Schweickert et al. 2009 | Hospital discharge | 29 (59%) | 19 (35%) | 0.02 |
| **SF-36 Physical Function Domain Score (PFS)*** | (i) | Morris et al. 2016**** | Hospital discharge | 38.3 (28.1) | 38.4 (27.8) | 0.97 |
|  |  |  | 2 month follow-up | 43.0 (26.9) | 47.4 (27.2) | 0.29 |
|  |  |  | 4 month follow-up | 47.2 (26.0) | 52.2 (26.0) | 0.22 |
|  |  |  | 6 month follow-up | 43.6 (27.7) | 55.9 (27.0) | 0.001 |
|  | (ii) | Denehy et al. 2013 | 3 month follow-up | 42.3 (12) | 39.9 (14.4) | - |
|  |  |  | 6 month follow-up | 42.4 (13.7) | 40.1 (14.7) | - |
|  |  |  | 12 month follow-up | 44 (11.2) | 41.4 (12.5) | - |
|  |  | Kayambu et al. 2015 | 6 month follow-up | 60 (29.4) | 81.8 (22.2) | 0.04 |
|  |  | Eggmann et al. 2018 | 6 month follow-up | 75 (50-85) | 75 (45-85) | 0.68 |
| **SF-36 Physical Health Component Summary Score (PCS)*** | (i) | Morris et al. 2016**** | Hospital discharge | 30.3 (9.7) | 30.2 (9.8) | 0.96 |
|  |  |  | 2 month follow-up | 32.2 (7.6) | 33.4 (9.9) | 0.43 |
|  |  |  | 4 month follow-up | 33.7 (10.3) | 36.0 (10.4) | 0.16 |
|  |  |  | 6 month follow-up | 33.5 (11.1) | 36.9 (10.9) | 0.05 |
|  | (ii) | Denehy et al. 2013 | 3 month follow-up | 42.1 (9.6) | 41 (11.4) | - |
|  |  |  | 6 month follow-up | 44.4 (10.7) | 41.6 (13.2) | - |
|  |  |  | 12 month follow-up | 46.2 (9.4) | 44.7 (10.9) | - |
|  |  | Eggmann et al. 2018 | 6 month follow-up | 42.7 (10.4) | 40.8 (11.1) | 0.52 |

**=Prioritized by ICU Experts as most clinically relevant priority outcomes; **=Physical therapy group / physical+cognitive therapy group; ***=mini-modified FIM; ****=Standard deviation calculated from 95% confidence interval; (i)=systematic early vs. late mobilization, (ii)=systematic early vs. standard early mobilization.*

*Figure 2: Fixed- and random-effects meta-analysis of the mean differences [95% confidence interval] in Physical Function in ICU Test (PFIT) Scores at ICU discharge between systematic early mobilization and standard early mobilization.*

Cognitive function and mental health

| **Outcome** | **Comparison** | **Study** | **Time point** | **Comparator (Mean (SD)/Median (IQR) or  n (%))** | **Intervention [Mean (SD)/Median (IQR) or  n (%))** | **Reported p-Value** |
| --- | --- | --- | --- | --- | --- | --- |
| **Delirium duration (in days)** | (ii) | Schweickert et al. 2009 | ICU discharge | 4 (2-7) | 2 (0-6) | 0.03 |
|  |  |  | Hospital discharge | 4 (2-8) | 2 (0-6) | 0.02 |
|  |  | Morris et al. 2016 | Hospital discharge | 1 (0-3) | 1 (0-4) | 0.43 |
| **Delirium-free days** | (ii) | Schaller et al. 2016 | Hospital discharge | 22 (15-25) | 25 (16-27) | 0.016 |
| **Mini-Mental State Exam (MMSE)** | (i) | Morris et al. 2016* | Hospital discharge | 25.1 (3.9) | 25.4 (3.8) | 0.55 |
|  |  |  | 2 month follow-up | 26.8 (3.8) | 26.7 (3.8) | 0.86 |
|  |  |  | 4 month follow-up | 27.2 (2.9) | 27.6 (2.8) | 0.37 |
|  |  |  | 6 month follow-up | 27.0 (2.8) | 27.6 (2.8) | 0.17 |
|  | (ii) | Brummel et al. 2014** | Hospital discharge | 25 (24-28) | 25.6 (23.2-27.8) /  28 (25.8-29.0) | 0.09 |
|  |  |  | 3 month follow-up | 28 (26.8-29) | 29 (27.0-30.0) /  29 (27.9-29.8) | 0.64 |
| **Hospital Anxiety and Depression Scale (HADS)** | (ii) | Kayambu et al. 2015 | ICU discharge | 8/19 (42.1%) | 6/16 (37.5%) | 0.09 |
|  |  | Hodgson et al. 2016 | 6 month follow-up | 11.3 (7.1) | 11.6 (9.1) | 0.91 |
| **SF-36 Mental Health Domain Score (MHS)** | (ii) | Denehy et al. 2013 | 3 month follow-up | 44.7 (14.0) | 45.67 (14.2) | - |
|  |  |  | 6 month follow-up | 45.6 (13.0) | 44.30 (14.8) | - |
|  |  |  | 12 month follow-up | 45.9 (16.5) | 46.69 (13.1) | - |
|  |  | Kayambu et al. 2015 | 6 month follow-up | 37.3 (7.4) | 38.6 (11.5) | 0.71 |
|  |  | Eggmann et al. 2018 | 6 month follow-up | 70 (64-76) | 84 (68-88) | 0.023 |
| **SF-36 Mental Health Component Summary Score (MCS)** | (i) | Morris et al. 2016* | Hospital discharge | 43.3 (11.0) | 43.6 (11.1) | 0.86 |
|  |  |  | 2 month follow-up | 46.2 (11.6) | 46.3 (12.0) | 0.96 |
|  |  |  | 4 month follow-up | 47.7 (11.0) | 47.8 (11.1) | 0.91 |
|  |  |  | 6 month follow-up | 46.4 (11.8) | 48.8 (11.6) | 0.19 |
|  | (ii) | Denehy et al. 2013 | 3 month follow-up | 46.3 (12) | 46.0 (13.9) | - |
|  |  |  | 6 month follow-up | 46.2 (12.9) | 45.8 (12.9) | - |
|  |  |  | 12 month follow-up | 44.7 (15.7) | 47.9 (12.3) | - |
|  |  | Eggmann et al. 2018 | 6 month follow-up | 45.2 (11.4) | 49.4 (10.3) | 0.15 |

**=Standard deviation calculated from 95% confidence interval; **=Physical therapy group / physical+cognitive therapy group; (i)=systematic early vs. late mobilization, (ii)=systematic early vs. standard early mobilization.*

*Figure 3: Fixed- and random-effects meta-analysis of the mean differences [95% confidence interval] in SF-36 Mental Health Component Summary Scores (MCS) 6 months between systematic early mobilization and late or standard early mobilization.*

Quality of Life

| **Outcome** | **Comparison** | **Study** | **Time point** | **Comparator (Mean (SD)/Median (IQR))** | **Intervention [Mean (SD)/Median (IQR))** | **Reported p-Value** |
| --- | --- | --- | --- | --- | --- | --- |
| **EQ-5D VAS** | (ii) | Brummel et al. 2014* | 3 month follow-up | 75 (61-86) | 80 (62-89) / 75 (54-80) | 0.44 |
|  |  | Hodgson et al. 2016 | 6 month follow-up | 68 (19) | 61 (19) | 0.25 |
| **SF-36 (overall score)** | (ii) | Schaller et al. 2016 | 3 month follow-up | 63.0 (19.9) | 61.3 (18.4) | 0.69 |

**=Physical therapy group / physical+cognitive therapy group; (ii)=systematic early vs. standard early mobilization.*

Mortality

| **Outcome** | **Comparison** | **Study** | **Comparator (Mean (SD)/Median (IQR) or  n (%))** | **Intervention [Mean (SD)/Median (IQR) or  n (%))** | **Reported p-Value** |
| --- | --- | --- | --- | --- | --- |
| **ICU mortality** | (ii) | Kayambu et al. 2015 | 1/24 (4.2%) | 3/26 (11.5%) | 0.34 |
|  |  | Hodgson et al. 2016 | 1/21 (4.8%) | 2/29 (6.9%) | 0.75 |
|  |  | Eggmann et al. 2018 | 10/57 (17.5%) | 9/58 (15.5%) | 0.77 |
|  | (iii) | Fischer et al. 2016 | 3/27 (11.1%) | 1/27 (3.7%) | - |
| **In-hospital mortality** | (i) | Schweickert et al. 2009 | 14/55 (25.5%) | 9/49 (18.4%) | 0.53 |
|  |  | Morris et al. 2016 | 18/150 (12.0%) | 18/150 (12.0%) | - |
|  | (ii) | Brummel et al. 2014* | 6/22 (27.3%) | 6/22 (27.3%) /  11/43 (25.6%) | - |
|  |  | Dong et al. 2014 | 3/30 (10.0%) | 2/30 (6.7%) | 1.0 |
|  |  | Dong et al. 2016 | 3/53 (5.7%) | 2/53 (3.8%) | 0.65 |
|  |  | Hodgson et al. 2016 | 1/21 (4.8%) | 2/29 (6.9%) | 0.75 |
|  |  | Schaller et al. 2016 | 8/96 (8.3%) | 17/104 (16.4%) | 0.09 |
|  |  | Eggmann et al. 2018 | 14/57 (24.6%) | 10/58 (17.2%) | 0.33 |
| **Mortality at 3 months** | (ii) | Denehy et al. 2013 | 13/76 (17.1%) | 10/74 (13.5%) | - |
|  |  | Brummel et al. 2014* | 9/22 (40.9%) | 7/22 (31.8%) /  16/43 (37.2%) | - |
|  |  | Kayambu et al. 2015 | 2/24 (8.3%) | 8/26 (30.8%) | 0.08 |
|  |  | Schaller et al. 2016 | 15/96 (15.6%) | 21/104 (20.2%) | 0.35 |
| **Mortality at 6 months** | (i) | Morris et al. 2016 | 33/150 (22.0%) | 33/150 (22.0%) | - |
|  | (ii) | Denehy et al. 2013 | 14/76 (18.4%) | 10/74 (13.5%) | - |
|  |  | Kayambu et al. 2015 | 4/24 (16.7%) | 12/26 (46.2%) | - |
|  |  | Eggmann et al. 2018 | 16/57 (28.1%) | 16/58 (27.6%) | 0.95 |
| **Mortality at 12 months** | (ii) | Denehy et al. 2013 | 19/76 (25.0%) | 13/74 (17.6%) | - |

**=Physical therapy group / physical+cognitive therapy group; (i)=systematic early vs. late mobilization, (ii)=systematic early vs. standard early mobilization; (iii)=systematic early vs. no mobilization.*

*Figure 4: Fixed- and random-effects meta-analysis of the risk ratios [95% confidence interval] for in-hospital mortality and 6-month mortality between systematic early mobilization and late or standard early mobilization.*

Additional Outcomes

| **Outcome** | **Comparison** | **Study** | **Comparator (Mean (SD)/Median (IQR))** | **Intervention [Mean (SD)/Median (IQR))** | **Reported p-Value** |
| --- | --- | --- | --- | --- | --- |
| **Length of ICU stay (ICU LOS; in days)** | (i) | Schweickert et al. 2009 | 7.9 (6.1-12.9) | 5.9 (4.5-13.2) | 0.08 |
|  |  | Morris et al. 2016 | 8 (4-13) | 7.5 (4-14) | 0.68 |
|  | (ii) | Dantas et al. 2012 | 21.4 (17.1) | 19.9 (11.7) | 0.77 |
|  |  | Denehy et al. 2013 | 7 (6-11) | 8 (6-12) | - |
|  |  | Brummel et al. 2014* | 4 (3.0-6.7) | 3.5 (2.3-7.2) / 5 (2.8-9.6) | 0.67 |
|  |  | Dong et al. 2014 | 15.2 (4.5) | 12.7 (4.1) | 0.01 |
|  |  | Kayambu et al. 2015 | 8.5 (3-36) | 12.0 (4-45) | 0.43 |
|  |  | Dong et al. 2016 | 18.3 (4.2) | 11.7 (3.2) | <0.01 |
|  |  | Hodgson et al. 2016 | 11 (8-19) | 9 (6-17) | 0.28 |
|  |  | Schaller et al. 2016 | 10 (6-15) | 7 (5-12) | 0.005 |
|  |  | Eggmann et al. 2018 | 6.6 (4.6-14.7) | 6.1 (4.0-12.3) | 0.57 |
|  | (iii) | Fischer et al. 2016 | 7 (range 3-213) | 6 (range 2-23) | 0.46 |
| **Length of hospital stay (hospital LOS; in days)** | (i) | Schweickert et al. 2009 | 12.9 (8.9-19.8) | 13.5 (8.0-23.1) | 0.93 |
|  |  | Morris et al. 2016 | 10 (7-16) | 10 (6-17) | 0.41 |
|  | (ii) | Dantas et al. 2012 | 39.7 (17.6) | 32.2 (16.4) | 0.25 |
|  |  | Denehy et al. 2013 | 20 (13.0-30.8) | 23.5 (16.0-41.5) | - |
|  |  | Brummel et al. 2014* | 8.6 (6.0-16.2) | 7.0 (5.0-10.5) / 7.9 (5.1-15.0) | 0.46 |
|  |  | Kayambu et al. 2015 | 45 (14-308) | 41 (9-158) | 0.8 |
|  |  | Dong et al. 2016 | 29.1 (4.6) | 22.0 (3.8) | <0.01 |
|  |  | Hodgson et al. 2016 | 29 (16-34) | 19 (14-30) | 0.33 |
|  |  | Schaller et al. 2016 | 21.5 (15-30) | 15 (11-27) | 0.011 |
|  |  | Eggmann et al. 2018 | 22.0 (15.0-39.2) | 25.9 (14.3-37.2) | 0.72 |
|  | (iii) | Fischer et al. 2016 | 19 (range 9-213) | 22 (range 4-84) | 0.6 |
| **Duration of mechanical ventilation (in days)** | (i) | Schweickert et al. 2009 | 6.1 (4-9.6) | 3.4 (2.3-7.3) | 0.02 |
|  | (ii) | Dantas et al. 2012 | 13.25 (13.5) | 10.86 (9.6) | 0.6 |
|  |  | Denehy et al. 2013** | 98 (47.5-160.5) | 105 (52.0-216.5) | - |
|  |  | Dong et al. 2014 | 7.3 (2.8) | 5.6 (2.1) | 0.005 |
|  |  | Kayambu et al. 2015 | 7 (2-30) | 8 (4-64) | 0.22 |
|  |  | Dong et al. 2016 | 13.9 (4.1) | 8.1 (3.3) | <0.01 |
|  |  | Hodgson et al. 2016 | 7.0 (5.0-12.0) | 5.4 (3.5-10.0) | 0.18 |
|  |  | Eggmann et al. 2018 | 5.0 (3.6-11.9) | 5.4 (3.3-12.9) | 0.83 |
|  | (iii) | Fischer et al. 2016 | 2 (range 1-15) | 2 (range 1-7) | - |
| **Ventilator-free days** | (i) | Schweickert et al. 2009 | 21.1 (0-23.8) | 23.5 (7.4-25.6) | 0.05 |
|  |  | Morris et al. 2016 | 24 (20-26) | 24 (19-26) | 0.59 |
|  | (ii) | Brummel et al. 2014* | 27.4 (0-29.2) | 27.1 (1.7-28.7) /  25.3 (0-28.9) | 0.81 |
|  |  | Kayambu et al. 2015 | 21 (0-26) | 20 (0-24) | 0.71 |
|  |  | Hodgson et al. 2016 | 17.1 (8.7) | 19.2 (7.4) | 0.4 |
|  |  | Schaller et al. 2016 | 22.5 (16-25) | 23 (18-25) | 0.31 |

**=Physical therapy group / physical+cognitive therapy group; **=in hours; (i)=systematic early vs. late mobilization, (ii)=systematic early vs. standard early mobilization; (iii)=systematic early vs. no mobilization.*

Safety outcomes

Information on safety and adverse events were available from nine out of twelve studies [1–9]. Schweickert et al. reported the occurrence of one event of oxygen desaturation and one inadvertent removal of an arterial catheter in the intervention group (less than 0.1% out of total 498 therapy sessions) [1]. Denehy et al.[2] reported no observed adverse events. Brummel et al. reported episodes of hypotension or tachycardia in 21 therapy sessions (4% in total 543 sessions) and one hypertensive urgency with acute backache. They reported no removal of endotracheal tubes or vascular catheters [3]. Dong et al. (2014) reported the occurrence of one episode of orthostatic hypotension without serious adverse effects [4]. Kayambu et al. reported no observed adverse events in more than 600 physiotherapy sessions in the intervention group (visual assessment of presented figure) [5]. Hodgson et al. reported that no serious adverse events occurred. They reported the occurrence of one episode of agitation in the intervention group and two episodes of agitation and one hypotensive episode in the comparator group [6]. Morris et al. reported a total of 4 severe adverse events (total 11 adverse events) in the intervention group and 3 severe adverse events (total 13 adverse events) in the comparator group [7]. Only one event in the intervention group was considered as potentially related to the early mobilization intervention and one was considered life-threatening. Schaller et al. reported 11 episodes of hypotension, two episodes of oxygen desaturation, one dislodgement of an arterial catheter and one dislodgement of a nasogastric tube in the intervention group. In comparison, they reported five episodes of hypotension, two episodes of oxygen desaturation and two occurrences of dislodgement of an arterial catheter in the comparator group [8]. They further reported 10 minor adverse events in the intervention group and one minor adverse event in the comparator group. Eggmann et al. reported one episode of oxygen desaturation in the intervention group (<0.1% out of 407 physiotherapy sessions), as well as one episode of oxygen desaturation and two episodes of unstable hemodynamics in the comparator group (both <0.1% in 377 sessions) [9]. No information regarding adverse effecdts was available from Dong et al. (2016) [10], Dantas et al. [11] and Fischer et al. [12].

References

1 Schweickert WD, Pohlman MC, Pohlman AS, *et al.* Early physical and occupational therapy in mechanically ventilated, critically ill patients: a randomised controlled trial. *The Lancet* 2009;**373**:1874–82. doi:10.1016/S0140-6736(09)60658-9

2 Denehy L, Skinner EH, Edbrooke L, *et al.* Exercise rehabilitation for patients with critical illness: a randomized controlled trial with 12 months of follow-up. *Crit Care* 2013;**17**:R156.

3 Brummel NE, Girard TD, Ely EW, *et al.* Feasibility and safety of early combined cognitive and physical therapy for critically ill medical and surgical patients: the Activity and Cognitive Therapy in ICU (ACT-ICU) trial. *Intensive Care Med* 2014;**40**:370–9. doi:10.1007/s00134-013-3136-0

4 Dong Z, Yu B, Sun Y, *et al.* Effects of early rehabilitation therapy on patients with mechanical ventilation. *World J Emerg Med* 2014;**5**:48–52. doi:10.5847/wjem.j.issn.1920-8642.2014.01.008

5 Kayambu G, Boots R, Paratz J. Early physical rehabilitation in intensive care patients with sepsis syndromes: a pilot randomised controlled trial. *Intensive Care Med* 2015;**41**:865–74. doi:10.1007/s00134-015-3763-8

6 Hodgson CL, Bailey M, Bellomo R, *et al.* A Binational Multicenter Pilot Feasibility Randomized Controlled Trial of Early Goal-Directed Mobilization in the ICU. *Crit Care Med* 2016;**44**:1145. doi:10.1097/CCM.0000000000001643

7 Morris PE, Berry MJ, Files DC, *et al.* Standardized Rehabilitation and Hospital Length of Stay Among Patients With Acute Respiratory Failure: A Randomized Clinical Trial. *JAMA* 2016;**315**:2694–702. doi:10.1001/jama.2016.7201

8 Schaller SJ, Anstey M, Blobner M, *et al.* Early, goal-directed mobilisation in the surgical intensive care unit: a randomised controlled trial. *The Lancet* 2016;**388**:1377–88. doi:10.1016/S0140-6736(16)31637-3

9 Eggmann S, Verra ML, Luder G, *et al.* Effects of early, combined endurance and resistance training in mechanically ventilated, critically ill patients: A randomised controlled trial. *PLoS ONE* 2018;**13**:1–19. doi:10.1371/journal.pone.0207428

10 Dong Z, Yu B, Zhang Q, *et al.* Early Rehabilitation Therapy Is Beneficial for Patients With Prolonged Mechanical Ventilation After Coronary Artery Bypass Surgery. *Int Heart J* 2016;**57**:241–6. doi:10.1536/ihj.15-316

11 Dantas CM, Silva PF dos S, Siqueira FHT de, *et al.* Influence of early mobilization on respiratory and peripheral muscle strength in critically ill patients. *Rev Bras Ter Intensiva* 2012;**24**:173–8. doi:10.1590/S0103-507X2012000200013

12 Fischer A, Spiegl M, Altmann K, *et al.* Muscle mass, strength and functional outcomes in critically ill patients after cardiothoracic surgery: does neuromuscular electrical stimulation help? The Catastim 2 randomized controlled trial. *Crit Care* 2016;**20**:30. doi:10.1186/s13054-016-1199-3
