# Supplementary material for: Systematic early versus late mobilization or standard early mobilization in mechanically ventilated adult ICU patients: systematic review and meta-analysis
Source: Crit Care. 2021 Jan 6;25:16. doi: 10.1186/s13054-020-03446-9 (PMC7789482; doi:10.1186/s13054-020-03446-9)
Supplement: Supplementary file 4 — Additional file 4. GRADE evidence profile. [file 13054_2020_3446_MOESM4_ESM.docx]

**Additional File 4**

**GRADE Evidence Profile Details**

| **Outcome** | **Certainty assessment** | | | | | | | **Certainty** | **Importance** |
| --- | --- | --- | --- | --- | --- | --- | --- | --- | --- |
| **Comparison** | **№ of studies** | **Study design** | **Risk of bias** | **Inconsistency** | **Indirectness** | **Imprecision** | **Other considerations** |  |  |
| **MRC Sum Score (MRC-SS), measured at ICU discharge** | | | | | | | | | |
| (i) systematic early vs. late mobilization | 1 | randomized trials | not serious | not serious | not serious | serious ^a^ | only one study contributing to results ^b^ | ⨁⨁◯◯ LOW | critical |
| (ii) systematic early vs. standard early mobilization | 4 | randomized trials | serious ^c^ | serious ^d^ | not serious | serious ^a^ | none | ⨁◯◯◯ VERY LOW | critical |
| **6-Minute Walking Test (6MWT), measured at various time points** | | | | | | | | | |
| (ii) systematic early vs. standard early mobilization | 2 | randomized trials | not serious ^f^ | not serious | not serious | serious ^a^ | mainly one study contributing to results ^g^ | ⨁⨁◯◯ LOW | critical |
| **Time to walking, measured during the hospital stay** | | | | | | | | | |
| (i) systematic early vs. late mobilization mobilization | 1 | randomized trials | not serious | not serious | not serious | serious ^a^ | only one study contributing to results ^b^ | ⨁⨁◯◯ LOW | critical |
| (ii) systematic early vs. standard early mobilization | 2 | randomized trials | serious ^c^ | not serious | not serious | serious ^a^ | mainly one study contributing to results ^h^ | ⨁◯◯◯ VERY LOW | critical |
| **Patients returning to independence from assistance, measured at hospital discharge** | | | | | | | | | |
| (i) systematic early vs. late mobilization | 1 | randomized trials | not serious | not serious | not serious | serious ^a^ | only one study contributing to results ^b^ | ⨁⨁◯◯ LOW | critical |

| **Outcome** | **Certainty assessment** | | | | | | | **Certainty** | **Importance** |
| --- | --- | --- | --- | --- | --- | --- | --- | --- | --- |
| **Comparison** | **№ of studies** | **Study design** | **Risk of bias** | **Inconsistency** | **Indirectness** | **Imprecision** | **Other considerations** |  |  |
| **SF-36 Physical Function Domain Score (PFS), measured 6 months after hospital discharge** | | | | | | | | | |
| (i) systematic early vs. late mobilization | 1 | randomized trials | serious ^c^ | not serious | not serious | serious ^a^ | only one study contributing to results ^b^ | ⨁◯◯◯ VERY LOW | critical |
| (ii) systematic early vs. standard early mobilization | 2 | randomized trials | serious ^c^ | serious ^d^ | not serious | serious ^a,e^ | none | ⨁◯◯◯ VERY LOW | critical |
| **SF-36 Physical Health Component Summary Score (PCS), measured 6 months after hospital discharge** | | | | | | | | | |
| (i) systematic early vs. late mobilization | 1 | randomized trials | serious ^c^ | not serious | not serious | serious ^a^ | only one study contributing to results ^b^ | ⨁◯◯◯ VERY LOW | critical |
| (ii) systematic early vs. standard early mobilization | 2 | randomized trials | serious ^c^ | not serious | not serious | very serious ^e^ | none | ⨁◯◯◯ VERY LOW | critical |
| **Patients developing ICUAW, measured at hospital discharge** | | | | | | | | | |
| (i) systematic early vs. late mobilization | 1 | randomized trials | not serious | not serious | not serious | serious ^a^ | only one study contributing to results ^b^ | ⨁⨁◯◯ LOW | critical |
| (ii) systematic early vs. standard early mobilization | 3 | randomized trials | serious ^c^ | not serious | not serious | very serious ^e^ | none | ⨁◯◯◯ VERY LOW | critical |

**Explanations**

a. Downgraded one point due to imprecision (defined as wide confidence intervals including no effect and/or low overall sample size (defined as <400 participants for continuous outcomes or below optimal information size for dichotomous outcomes)).

b. Downgraded one point due to only one study contributing to outcome.

c. Downgraded one point as majority of studies judged as of overall poor quality regarding risk of bias.

d. Downgraded one point due to presence of substantial unexplained heterogeneity.

e. Downgraded two points due to high imprecision (wide confidence intervals for absolute effects including important harm and low overall sample size (see definition above)).

f. Not downgraded as we judged the risk of bias of studies contributing data as not relevant for outcome.

g. Downgraded one point due to only one study contributing to outcome (change from baseline deemed most important aspect of outcome).

h. Downgraded one point due to only one study contributing to outcome (the second study barely contributed data (n=3).
